# Blood coagulation factor XII drives adaptive immunity during neuroinflammation via CD87-mediated modulation of dendritic cells

**DOI:** 10.1038/ncomms11626

**Published:** 2016-05-18

**Authors:** Kerstin Göbel, Susann Pankratz, Chloi-Magdalini Asaridou, Alexander M. Herrmann, Stefan Bittner, Monika Merker, Tobias Ruck, Sarah Glumm, Friederike Langhauser, Peter Kraft, Thorsten F. Krug, Johanna Breuer, Martin Herold, Catharina C. Gross, Denise Beckmann, Adelheid Korb-Pap, Michael K. Schuhmann, Stefanie Kuerten, Ioannis Mitroulis, Clemens Ruppert, Marc W. Nolte, Con Panousis, Luisa Klotz, Beate Kehrel, Thomas Korn, Harald F. Langer, Thomas Pap, Bernhard Nieswandt, Heinz Wiendl, Triantafyllos Chavakis, Christoph Kleinschnitz, Sven G. Meuth

**Affiliations:** 1 Department of Neurology, Clinic of Neurology and Institute for Translational Neurology, University of Münster, 48149 Münster, Germany; 2 Department of Neurology, University Medical Center of the Johannes Gutenberg-University, 55131 Mainz, Germany; 3 Department of Neurology, University Hospital Würzburg, 97080 Würzburg, Germany; 4 Institute of Experimental Musculoskeletal Medicine, University Hospital Münster, 48149 Münster, Germany; 5 Department of Anatomy and Cell Biology, University of Würzburg, 97070 Würzburg, Germany; 6 Department of Clinical Pathobiochemistry and Institute for Clinical Chemistry and Laboratory Medicine, University Clinic Carl Gustav Carus, Technische Universität of Dresden, 01307 Dresden, Germany; 7 Department of Internal Medicine, Universities of Giessen & Marburg Lung Center (UGMLC)/Member of the German Center for Lung Research (DZL), Justus-Liebig University, 35392 Giessen, Germany; 8 CSL Behring GmbH, 35041 Marburg, Germany; 9 CSL Limited, Bio21 Institute, Parkville, Victoria 3010, Australia; 10 Department of Anesthesiology, Intensive Care and Pain Medicine, Experimental and Clinical Hemostasis, University of Münster, 48149 Münster, Germany; 11 Department of Neurology, Klinikum rechts der Isar, Technical University of Munich, 81675 Munich, Germany; 12 Department of Cardiology and Cardiovascular Medicine, Section for Cardioimmunology, University Clinic of Tübingen, 72076 Tübingen, Germany; 13 Rudolf Virchow Center, Deutsche Forschungsgemeinschaft Research Center for Experimental Biomedicine, University of Würzburg, 97080 Würzburg, Germany; 14 Department of Neurology, University Hospital Essen, 45147 Essen, Germany

## Abstract

Aberrant immune responses represent the underlying cause of central nervous system (CNS) autoimmunity, including multiple sclerosis (MS). Recent evidence implicated the crosstalk between coagulation and immunity in CNS autoimmunity. Here we identify coagulation factor XII (FXII), the initiator of the intrinsic coagulation cascade and the kallikrein–kinin system, as a specific immune cell modulator. High levels of FXII activity are present in the plasma of MS patients during relapse. Deficiency or pharmacologic blockade of FXII renders mice less susceptible to experimental autoimmune encephalomyelitis (a model of MS) and is accompanied by reduced numbers of interleukin-17A-producing T cells. Immune activation by FXII is mediated by dendritic cells in a CD87-dependent manner and involves alterations in intracellular cyclic AMP formation. Our study demonstrates that a member of the plasmatic coagulation cascade is a key mediator of autoimmunity. FXII inhibition may provide a strategy to combat MS and other immune-related disorders.

## Abstract (web summary)

Factor XII initiates the intrinsic blood coagulation cascade and the kinin system. Here the authors show that Factor XII is elevated in the blood of multiple sclerosis patients, activates dendritic cells via CD87 and cAMP, and its blockade inhibits immunopathology in a mouse model of the disease.

## Graphical abstract

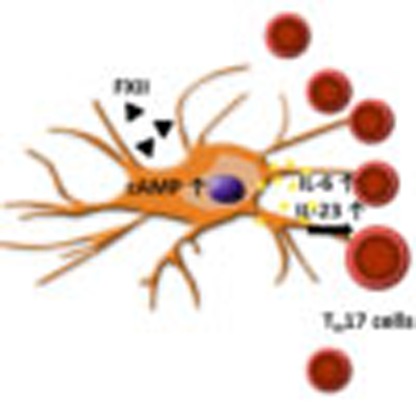

Autoimmune diseases of the central nervous system (CNS), such as multiple sclerosis (MS), are mediated by the intimate interplay of many cellular and molecular immune components^1^,^2^. It is widely accepted that autoreactive T cells generated in the periphery migrate across the blood–brain barrier (BBB), inducing disseminated inflammatory lesions within the brain parenchyma, leading to demyelination. Recent studies suggest that both interferon (IFN)-γ- and interleukin (IL)-17A-producing effector T-helper cells (T_H_1 and T_H_17, respectively) contribute to inflammation and tissue damage in the course of CNS autoimmunity^3^,^4^,^5^. Interaction of T cells with dendritic cells (DCs), professional antigen-presenting cells (APCs), is crucial for T-cell differentiation^6^,^7^. Accumulation of effector T cells in human brain lesions and subsequent increased expression of cell-specific signature cytokines in peripheral blood mononuclear cells (PBMCs) of patients also indicate a role of autoreactive T cells in human MS^8^,^9^.

More recent evidence suggests that other factors not traditionally considered components of the immune system might also be involved in MS pathophysiology. In particular, blood coagulation constituents, such as platelets, are thought to contribute to experimental autoimmune encephalomyelitis (EAE), the mouse model of human MS^10^. Moreover, deposition of plasmatic coagulation factors, such as fibrinogen, has been described in human MS lesions^11^,^12^,^13^, and tissue factor and protein C inhibitor have been identified within chronic active MS plaques^14^.

Although findings indicate a role of the extrinsic coagulation system in EAE and MS, the function of the intrinsic coagulation system remains unknown. The initiator of intrinsic coagulation is factor XII (FXII; Hageman factor)^15^. FXII activation occurs through the contact with negatively charged surfaces^16^, resulting in activation of the intrinsic blood coagulation system and subsequently fibrin clot formation^15^,^17^. FXII also triggers the proinflammatory kallikrein–kinin system (KKS), which consists of several sequentially linked serine proteases, with the peptide hormone bradykinin (BK) being the end product. In addition, FXII may interact with cell-surface-associated receptors, such as the urokinase plasminogen activator receptor (also designated CD87)^18^.

As FXII is at the interface between inflammation and coagulation, and has recently been identified as a major driving force during ischaemic neurodegeneration^19^, we therefore investigated its role in autoimmunity and the potential underlying mechanisms of action. Moreover, we assessed FXII as a therapeutic target in different EAE models. We showed that FXII drives pathologic adaptive immune reactions via CD87-mediated modulation of DC.

## Results

### FXII-deficient mice are less susceptible to CNS inflammation

**Figure 1:**
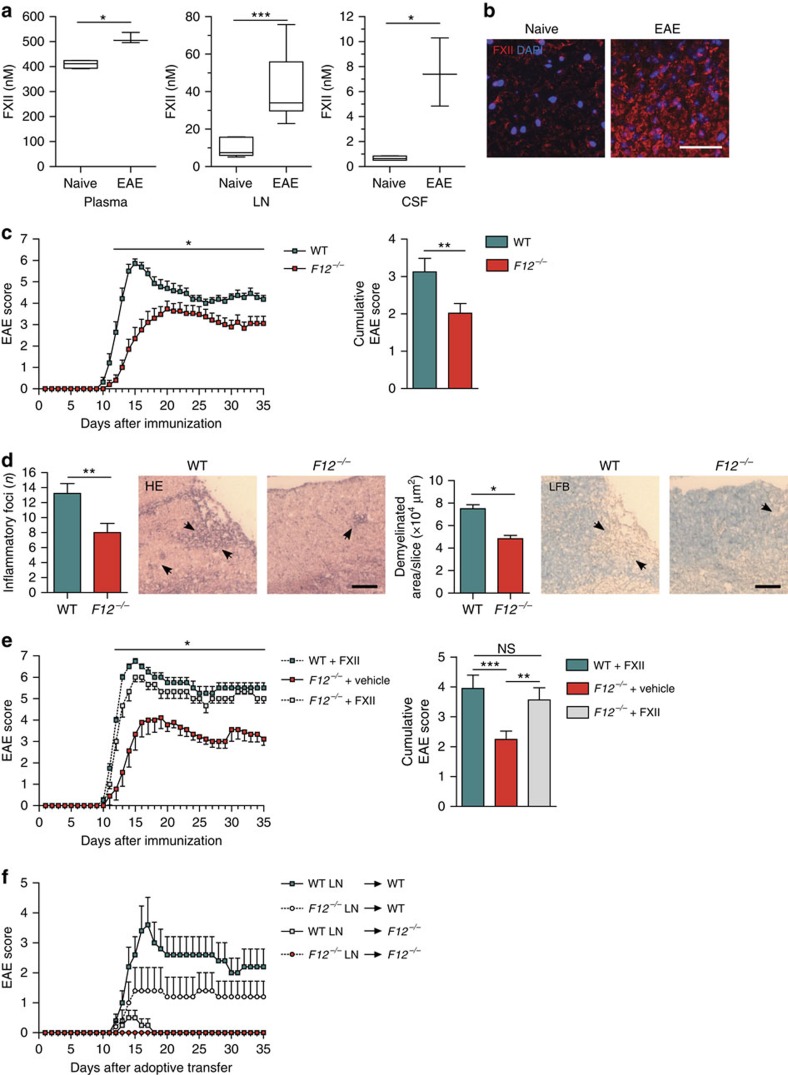
FXII-deficient animals are less susceptible to neuroinflammation. (**a**) FXII levels in plasma, LN and CSF of naive and active EAE-induced WT animals at *d*_max_ (day 16) were analysed by ELISA. Data are given as mean±s.e.m. from three independent experiments, each with five animals per group (Student's *t*-test). (**b**) Histological analysis of spinal cord sections from the lumbar region of MOG_35–55_-immunized WT animals at *d*_max_ is shown. Sections were stained for FXII (red) and nucleus (4,6-diamidino-2-phenylindole (DAPI), blue). Scale bar, 50 μm. (**c**) Active EAE was induced in WT and *F12*^−/−^ mice. Data are mean clinical scores±s.e.m. and mean cumulative scores±s.e.m. of WT and *F12*^−/−^ animals from three independent experiments (non-parametric Mann–Whitney *U*-test). For detailed animal numbers and additional information, see Supplementary Table 1. (**d**) Identification of inflammatory foci (left panels) and demyelination (right panels) in spinal cord sections from the lumbar region of WT and *F12*^−/−^ animals by haematoxylin and eosin (HE) or Luxol fast blue (LFB) staining. Scale bars, 100 μm. Quantification and representative histological sections from *d*_max_ of EAE are shown. Data are presented as mean±s.e.m. (*n*=3 slices of six mice per group, Student's *t*-test). Arrows indicate inflammation or demyelinated areas, respectively. (**e**) Mean clinical scores and mean cumulative scores±s.e.m. over time of WT or *F12*^−/−^ mice treated daily with intravenous injections of FXII (200 mg kg^−1^ body weight) or corresponding vehicle since MOG_35–55_ immunization are shown. (**f**) EAE development in WT and *F12*^−/−^ mice after adoptive transfer of encephalitogenic LN cells is shown. LN cells were isolated from WT or *F12*^−/−^ mice on day 12 post immunization and restimulated *in vitro* with 10 μg ml^−1^ MOG_35–55_ and 0.5 ng ml^−1^ interleukin-12. After 72 h, 8.4 × 10^6^ LN cells were either transferred into WT or *F12*^−/−^ recipient mice. Mean clinical scores±s.e.m. over time of three independent experiments are given (non-parametric Mann–Whitney *U*-test). *P<0.05, **P<0.01, ***P<0.001; NS, not significant.

To assess whether FXII is relevant during CNS autoimmunity *in vivo*, we first analysed the plasma, lymph nodes (LNs) and the inflamed CNS of myelin oligodendrocyte glycoprotein 35–55 (MOG_35–55_)-immunized wild-type (WT) mice. Interestingly, FXII levels were significantly increased in the plasma and LN on disease maximum of EAE (Fig. 1a). Despite these peripheral alterations, extensive FXII depositions could also be found in the inflamed CNS of immunized mice (Fig. 1a,b), implying a potential contribution of FXII to EAE pathology.

To this end, we subjected FXII-deficient (*F12*^−/−^) mice to EAE. *F12*^−/−^ and WT control mice were immunized with MOG_35–55_, and clinical scores were evaluated daily over a 35-day period. FXII deficiency was associated with later disease onset and reduced maximum disease severity (*d*_max_) (Fig. 1c; Supplementary Table 1). Inflammatory infiltrates and demyelination were less prevalent in MOG_35–55_-immunized *F12*^−/−^ mice compared with WT mice (Fig. 1d). While the distribution of different immune cell subsets in spleens and LNs from *F12*^−/−^ and WT mice was only slightly shifted under basal (that is, non-immunized, reduced CD11c^+^ cells, increased forkhead box P3 (FoxP3) cells) and MOG_35–55_-immunized conditions (Supplementary Table 2; Supplementary Fig. 1), FXII deficiency was associated with lower relative numbers of CD4^+^ and CD8^+^ T cells as well as CD11b^+^ and CD11c^+^ cells in the CNS of EAE mice at *d*_max_ (Supplementary Table 2; Supplementary Fig. 2).

Importantly, reconstitution of *F12*^−/−^ animals with human FXII fully restored the susceptibility to EAE (Fig. 1e), indicating that the protective phenotype in EAE, due to FXII deficiency, can be specifically attributed to the absence of FXII. To further test whether FXII is involved in the priming or effector phase of EAE, we performed adoptive transfer (AT)-EAE. Interestingly, WT and *F12*^−/−^ animals that received *F12*^−/−^ LN cells developed almost no signs of EAE. Similarly, *F12*^−/−^ mice receiving LN cells from WT or *F12*^−/−^ mice were likewise protected from AT-EAE, indicating that FXII impacts priming as well as the effector mechanisms within the inflamed CNS (Fig. 1f).

### FXII shifts immune cells towards a T_H_17 pattern

**Figure 2:**
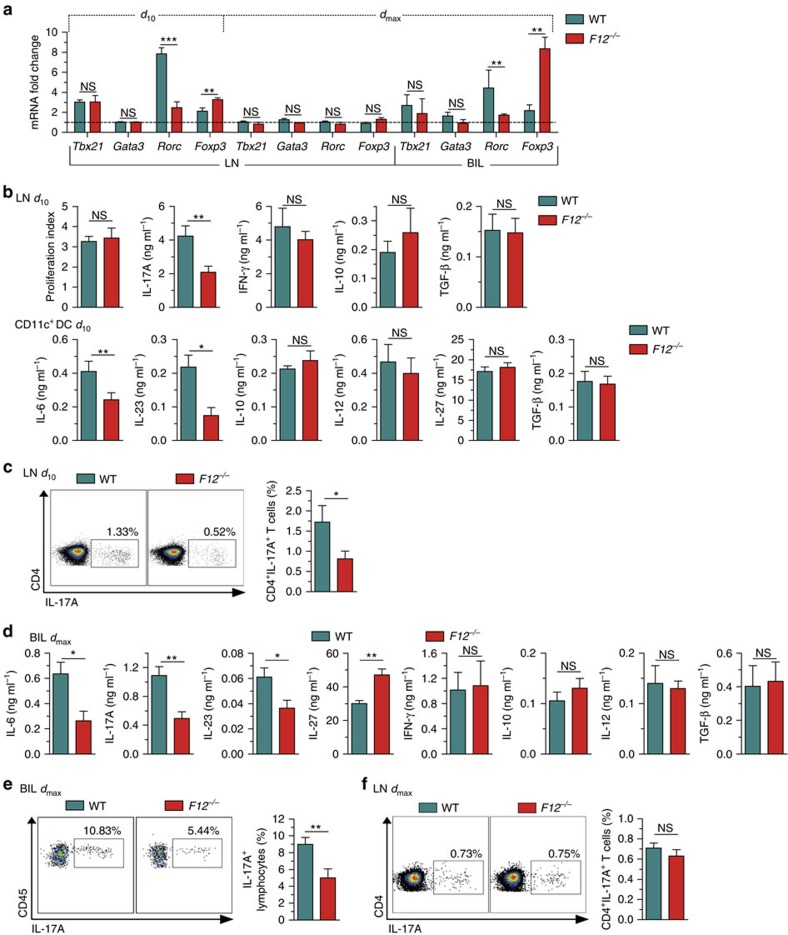
Factor XII deficiency alters T-cell differentiation. (**a**) *Tbx21*, *Gata3*, *Rorc* and *Foxp3* expression from LN cells at day 10 (*d*_10_) or *d*_max_ as well as from brain-infiltrating leukocytes (BILs) at *d*_max_ after MOG_35–55_ immunization is determined by real-time reverse transcription–PCR using 18S rRNA for normalization. Data (mean±s.e.m. of five experiments) are given as fold change in normalized gene expression in animals relative to WT controls. (**b**) At *d*_10_ after MOG_35–55_ immunization, proliferation and cytokine production by CD4^+^ T cells purified from LN and restimulated with 10 μg ml^−1^ MOG_35–55_ and irradiated (35 Gy) antigen-presenting cells *in vitro* for 48 h (upper panels), and by CD11c^+^ DCs purified from spleens and incubated with 1 μg ml^−1^ LPS *in vitro* for 48 h (lower panels) are shown. (**c**) Mononuclear cells were isolated from the LN of WT and *F12*^−/−^ animals at *d*_10_ post induction of EAE. Cells were polyclonal restimulated *in vitro*, stained with anti-CD3 and anti-CD4, fixed and permeabilized, stained intracellularly with anti-IL-17A and analysed by flow cytometry for the percentage of IL-17A-producing CD4^+^ T cells. (**d**) Cytokine production by purified BILs from MOG_35–55_-immunized WT or *F12*^−/−^ mice at *d*_max_ after restimulation with 10 μg ml^−1^ MOG_35–55_ for 48 h. (**e**,**f**) BILs and LNs were isolated from WT and *F12*^−/−^ animals at *d*_max_ post induction of EAE and polyclonal restimulated *in vitro*. For the detection of the percentage of IL-17A-producing lymphocytes (CD45^high^CD11b^neg^ cells or CD4^+^CD3^+^ T cells), BILs or LNs were stained with anti-CD11b and anti-CD45, or anti-CD3 and anti-CD4, fixed and permeabilized, stained intracellularly with anti-IL-17A and analysed by flow cytometry. In **b**–**f**, data are given as means±s.e.m. of three independent experiments, each performed in triplicate. For **c**,**e** and **f**, representative dot plots for IL-17A expression are shown. For **a**–**f**, non-parametric Mann–Whitney *U*-test. *P<0.05, **P<0.01, ***P<0.001; NS, not significant.

We next evaluated the differentiation of immune cells on MOG_35–55_ immunization in an FXII-deficient or -proficient setting. To this end, active EAE was induced in WT and *F12*^−/−^ animals. At day 10 (priming phase) and *d*_max_ (day 16) of EAE, the amount of several transcription factor genes was determined in CD4^+^ T cells obtained from the LN or CNS infiltrates by real-time reverse transcription–PCR (rRT–PCR). CD4^+^ T cells of *F12*^−/−^ animals displayed no significant difference in *Tbx21* (coding for Tbet; a T_H_1 marker) and in *Gata3* transcripts (a T_H_2 marker), but a significant decrease in retinoic acid receptor-related orphan receptor C (*Rorc*) transcripts (encoding the T_H_17 specification marker) and an increase in *Foxp3* transcripts (a marker of regulatory T cells (T_reg_)) at day 10 (Fig. 2a). No significant changes were observed for any of these transcripts in CD4^+^ T cells at *d*_max_ (Fig. 2a). In line with the findings in the preclinical phase of EAE, brain-infiltrating lymphocytes (BILs) of *F12*^−/−^ animals displayed a similar pattern of *Tbx21*, *Gata3*, *Rorc* and *Foxp3* transcripts at *d*_max_, as already shown for CD4^+^ T cells at day 10 (Fig. 2a). These data suggest a FXII-induced shift to a T_H_17 signature in response to MOG_35–55_ immunization.

While proliferation of CD4^+^ T cells was unchanged in *F12*^−/−^ mice compared with WT animals, FXII favoured T_H_17 cell emergence as indicated by the cytokine profile (low IL-17A, unaltered IFN-γ, IL-10 and transforming growth factor (TGF)-β from CD4^+^ T cells within LN, and low IL-6, IL-23, unaltered IL-10, IL-12, IL-27 and TGF-β from CD11c^+^ DC; Fig. 2b) and by the number of IL-17A-producing CD4^+^ T cells obtained from LNs, as determined by flow cytometry in the preclinical phase (day 10; Fig. 2c). The same signature could be found in the CNS of EAE mice on *d*_max_ (low IL-6, IL-17A, IL-23, increased IL-27, unaltered IFN-γ, IL-10, IL-12, TGF-β and increased number of IL-17A-producing lymphocytes, Fig. 2d,e), while peripheral changes were already lost (unaltered number of IL-17A-producing cells, Fig. 2f). Notably, the amount of IL-17A-producing MOG_35–55_-activated CD4^+^CD40 ligand (CD40L)^+^ T cells obtained from draining LN was reduced in *F12*^*−/−*^ mice, as compared with WT animals, while the number of IFN-γ-producing CD4^+^CD40L^+^ T cells was unaltered, suggesting that antigen-specific sensitization and priming of T_H_17 cells is impaired in FXII deficiency (Supplementary Fig. 3).

### FXII worsens EAE independently of the coagulation and KKS

FXII can initiate both the proinflammatory KKS, resulting in BK release, and the intrinsic coagulation cascade, leading to fibrin formation^20^. Although basal serum BK levels were lower when FXII was absent, cerebrospinal fluid (CSF) BK levels of immunized *F12*^−/−^ animals were indistinguishable from those of immunized WT mice (Supplementary Fig. 4a). Accordingly, BK receptor 1 (B1R) or B2R expression levels in the inflamed spinal cords from *F12*^−/−^ and WT mice did not differ on EAE induction (Supplementary Fig. 4b). We recently showed that blockade of B1R on endothelial cells reduces immune cell invasion across the activated BBB in EAE^21^. We therefore analysed the impact of FXII deficiency on the migratory capacity of LN-derived immune cells in an *in vitro* transmigration assay, using murine brain microvascular endothelial cells (MBMECs) from B1R-deficient or WT mice. The number of immune cells crossing the MBMEC barrier did not differ significantly in either group (Supplementary Fig. 4c).

Western blot analysis revealed that the amount of fibrin/fibrinogen detectable in the CNS was similar between WT and *F12*^−/−^ mice at *d*_max_ (Supplementary Fig. 4d). That the effects of FXII in EAE were not mediated by the intrinsic coagulation system was corroborated by the fact that deficiency of FXI, the primary substrate of activated FXII during thrombus formation, did not alter the clinical course, demyelination, immune cell infiltration or cytokine levels in the course of EAE (Supplementary Fig. 4e–g). Overall, these findings indicate that the detrimental effects of FXII in autoimmune CNS inflammation cannot be attributed to the KKS or plasmatic coagulation cascade activation.

Another pathway, which is triggered by FXII, is the complement system^15^. Modulation of certain components of the complement system, such as C3a or C5a, has been shown to be relevant in EAE^22^. However, C5a serum levels were not significantly different between WT and *F12*^−/−^ animals (Supplementary Fig. 4h).

### FXII is pivotal for DC to orchestrate T-cell differentiation

**Figure 3:**
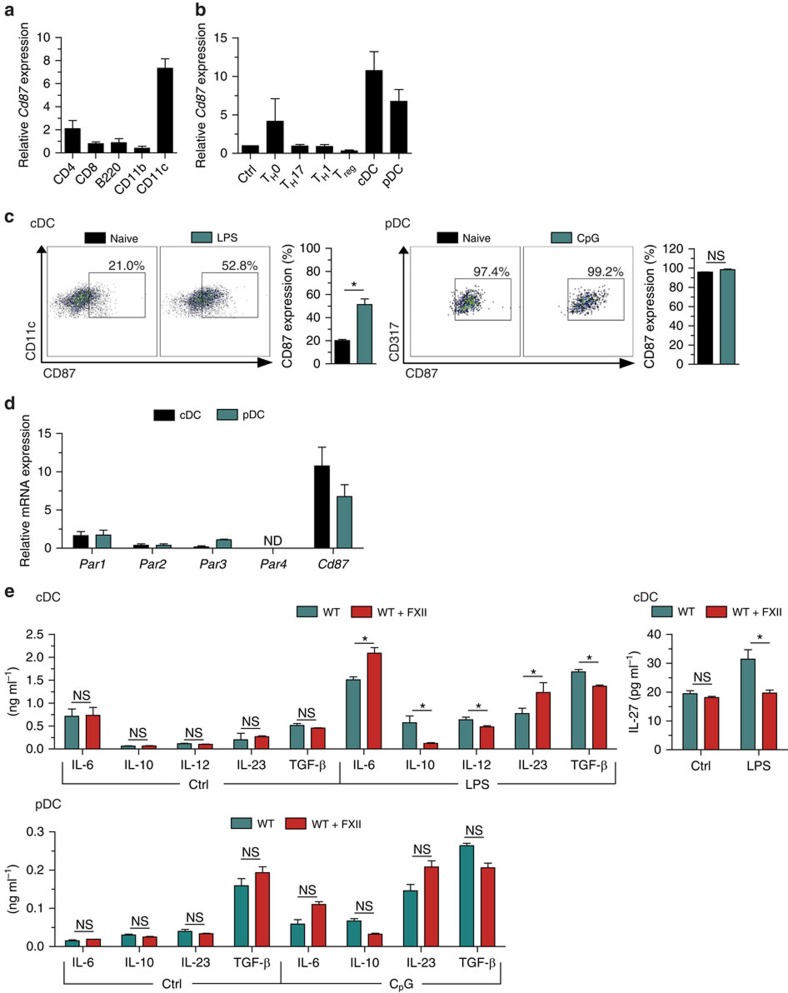
FXII favours the emergence of T_H_17 cells via DC. (**a**) Real-time reverse transcription–PCR (rRT–PCR) analyses for *Cd87* gene expression in CD4^+^, CD8^+^ and B220^+^ cells isolated from lymphocytes as well as in CD11b^+^ and CD11c^+^ cells isolated from spleens of naive WT mice. (**b**) rRT–PCR analyses for *Cd87* gene expression in CD4^+^CD25^−^ (Crtl) and CD4^+^CD25^+^ (T_reg_) cells isolated from lymph nodes under basal conditions or after a 48-h incubation with antibodies against CD3 and CD28 under neutral conditions (T_H_0) or in the presence of the appropriate cytokine and neutralizing antibody mixtures for differentiation into T_H_1 or T_H_17 cells as well as in splenic cDCs or pDCs. (**c**) Flow cytometry analysis for CD87 expression in cDCs (stained with CD11c, left panel) and pDCs (stained with CD317, right panel) isolated from the spleen under basal conditions or after a 24-h stimulation with 1 μg ml^−1^ LPS or 10 μg ml^−1^ CpG oligodeoxynucleotide 1,826, respectively. For cDCs and pDCs, representative dot plots for CD87 expression are shown. (**d**) rRT–PCR analyses for *Par1*, *Par2*, *Par3*, *Par4* and *Cd87* expression in cDCs and pDCs isolated from the spleen. (**e**) Splenic cDCs and pDCs from WT animals were incubated with medium only (Ctrl) or stimulated with 1 μg ml^−1^ LPS (for cDCs) or with 10 μg ml^−1^ CpG oligodeoxynucleotide 1,826 (for pDCs) in the absence or presence of 60 nM FXII, respectively. After 48 h, cytokine concentrations were measured in culture supernatants. In **a**,**b** and **d**, data are given as mean±s.e.m. of three independent experiments and presented as fold change in transcript expression relative to 18S rRNA. In **c** and **e**, data are given as mean±s.e.m. of three independent experiments, each performed in triplicate (non-parametric Mann–Whitney *U*-test). *P<0.05; ND, not detected; NS, not significant.

FXII can directly act on cells by binding to domain 2 of the glycoprotein CD87 (refs 16, 18). Hence, we evaluated *Cd87* expression in different immune cell subtypes isolated from the LN or spleen of naive WT mice by rRT–PCR (Fig. 3a). Interestingly, *Cd87* expression was prominent in CD11c^+^ DC, identifying those cells as a potential target of FXII. Naive CD4^+^ T-cell activation under T_H_1-, T_H_17- and inducible T_reg_ (iT_reg_)-favouring conditions did not lead to substantial CD87 upregulation (Fig. 3b). To search for CD87^+^ DC subsets, we purified conventional DCs (cDCs) and plasmacytoid DCs (pDCs) from WT mouse spleens. Both cell types expressed high levels of CD87 at both the messenger RNA and protein levels (Fig. 3b,c). Further protease-activated receptors (PAR), such as PAR1–4, are also able to interact with coagulation factors^23^. However, in contrast to CD87, cDC and pDC expressed only low numbers of *Par1–4* messenger RNA transcripts (Fig. 3d).

**Figure 4:**
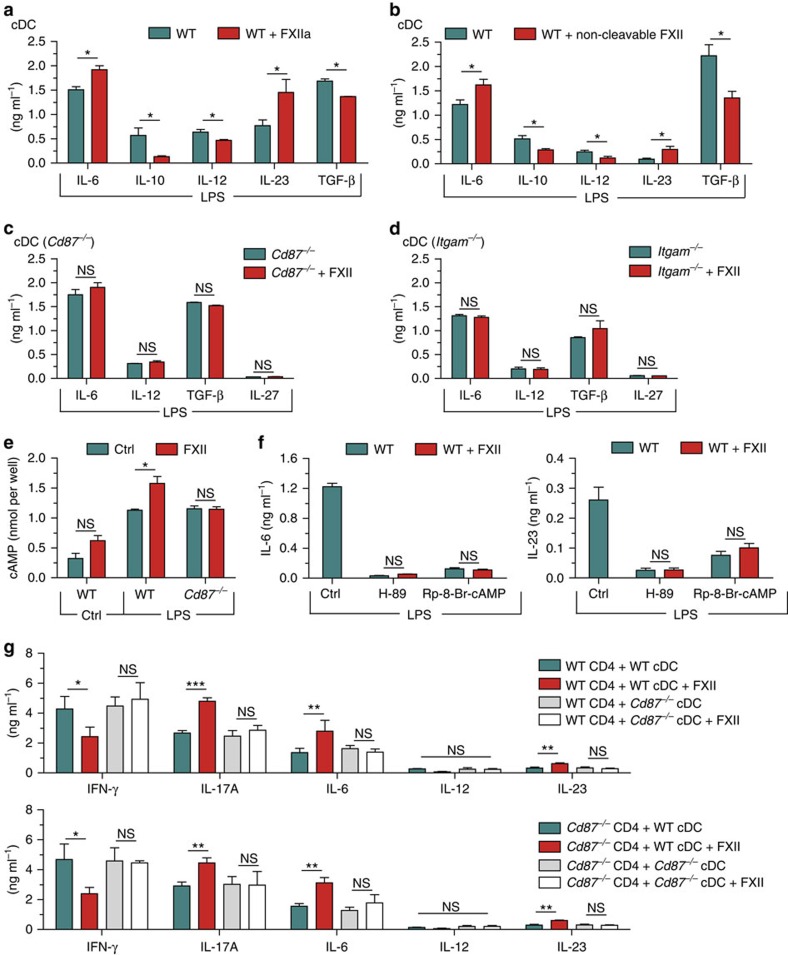
FXII controls cytokine production via CD87. (**a**) Splenic cDCs from naive WT animals were stimulated with 1 μg ml^−1^ LPS in the absence and presence of 60 nM activated FXII (FXIIa). After 48 h, cytokine concentrations were measured in culture supernatants by ELISA. (**b**) Cytokine production of splenic cDCs from WT animals stimulated with 1 μg ml^−1^ LPS in the absence and presence of 60 nM non-cleavable FXII. After 48 h, cytokine concentrations were determined in culture supernatants by ELISA. (**c**) Cytokine production of splenic cDCs from CD87-deficient (*Cd87*^−/−^) mice stimulated with 1 μg ml^−1^ LPS alone or in the absence and presence of 60 nM FXII for 48 h. (**d**) Cytokine production of splenic cDCs from CD11b-deficient (*Itgam*^−/−^) animals stimulated with 1 μg ml^−1^ LPS in the absence and presence of 60 nM FXII for 48 h. (**e**) Cytosolic cAMP formation measured in cDCs from WT or *Cd87*^−/−^ mice that were incubated with medium only (Ctrl) or stimulated with 1 μg ml^−1^ LPS for 10 min in the absence (Ctrl) or presence of 60 nM FXII. (**f**) Cytokine concentrations of IL-6 (left panel) and IL-23 (right panel) in the supernatants of WT cDCs stimulated with 1 μg ml^−1^ LPS or 60 nM FXII for 48 h in the presence of protein kinase A inhibitors (3 μM H-89 and 100 μM Rp-8-Br-cAMP). (**g**) Cytokine concentrations of IFN-γ, IL-17A, IL-6, IL-12 and IL-23 were measured in the supernatants from co-cultures of CD4^+^ T lymphocytes from WT (upper panel) or *Cd87*^−/−^ (lower panel) that were polyclonal activated together with cDCs from WT and *Cd87*^−/−^ in the absence or presence of 60 nM FXII. In **a**–**g**, data are given as means±s.e.m. of three independent experiments, each performed in duplicate (non-parametric Mann–Whitney *U*-test). *P<0.05, **P<0.01, ***P<0.001; NS, not significant.

We examined potential immunoregulatory functions of FXII pertinent to the interaction between CD4^+^ T cells and DC using different co-culture approaches. No significant differences in T-cell proliferation and cytokine production of IFN-γ and IL-17A were observed when incubating CD4^+^ T cells with FXII or vehicle (Supplementary Fig. 5a). In addition, T_H_1, T_H_17 or Foxp3 induction in CD4^+^ T cells cultivated under T_H_1-, T_H_17- and iT_reg_-inducing conditions, respectively, was not significantly affected by the presence of FXII (Supplementary Fig. 5b). In contrast, FXII-treated cDCs produced higher amounts of IL-6 in response to lipopolysaccharide (LPS), as compared with control conditions, while the levels of IL-10 and IL-12 were significantly reduced (Fig. 3e). In line with the finding for IL-6, the amount of IL-23 was also significantly higher, whereas the levels of TGF-β and IL-27 were reduced in the presence of FXII compared with controls (Fig. 3e). However, treatment of pDCs with FXII alone or together with CpG oligodeoxynucleotide 1,826 (CpG) did not lead to a significant shift in IL-6, IL-10, IL-23 or TGF-β production (Fig. 3e). For cDCs, similar results were obtained using FXIIa, the active form of FXII (Fig. 4a) and a non-cleavable FXII (Fig. 4b), indicating that both the active and inactive forms of FXII can modulate cDC function. Notably, immature and mature DCs did not differ significantly in terms of expression of the different surface markers in *F12*^−/−^ mice compared with WT controls or after exposure to FXII (Supplementary Figs 6 and 7).

To address the hypothesis that FXII modulates cDC function via CD87, cDCs of CD87-deficient (*Cd87*^−/−^) mice were treated with FXII or vehicle in the presence of LPS. In this scenario, cDC cytokine levels of IL-6, IL-12, TGF-β and IL-27 were not affected by FXII, indicating that CD87 on DCs is the main receptor mediating the effects of FXII (Fig. 4c). However, as CD87 is a glycoprotein without an intracellular domain and cannot induce intracellular signalling by itself, we sought to determine whether further transmembrane receptors are necessary to initiate intracellular signalling. Previous evidence has suggested that CD87 may form lateral complexes with β_2_-integrins, such as Mac-1 integrin (also designated CD11b/CD18), which could thereby serve as mediators of CD87-triggered signalling events^24^,^25^. Thus, we engaged CD11b-deficient (*Itgam*^−/−^) cDCs, which we treated with FXII or vehicle in the presence of LPS. FXII failed to induce changes in the production of cytokines in CD11b-deficient cDCs, indicating that the ligation of CD87 by FXII induces intracellular signalling in a CD11b-dependent manner (Fig. 4d).

Pathways inducing enhanced cyclic AMP (cAMP) formation in DCs are triggered by LPS and enhance cytokines associated with T_H_17 responses, such as IL-6 and IL-23, while decreasing T_H_1-associated cytokines, such as IL-12 and IL-27 (refs 26, 27). We therefore challenged WT cDCs with LPS in the presence and absence of FXII. Although FXII had only a slight effect on basal cAMP levels, it further increased the intracellular cAMP induced in cDCs by LPS (Fig. 4e). This effect was again CD87 dependent, as FXII failed to alter intracellular cAMP levels in *Cd87*-deficient DCs (Fig. 4e). Furthermore, production of T_H_17-associated cytokines was also reduced by treating WT cDCs with FXII in combination with different protein kinase A signalling inhibitors (Fig. 4f), indicating no significant role of protein kinase A-independent pathways of cAMP.

**Figure 5:**
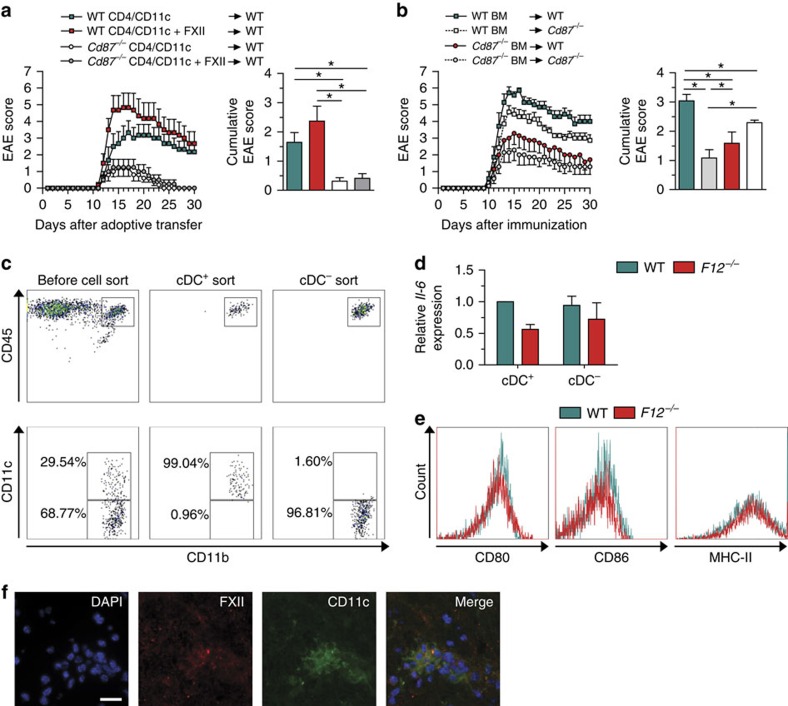
FXII influences DCs in the CNS. (**a**) EAE development is shown in WT mice after adoptive transfer of CD4^+^ lymphocytes and CD11c^+^ cells isolated from WT or *Cd87*^−/−^ mice on day 12 post immunization and restimulated with 10 μg ml^−1^ MOG_35–55_ and 0.5 ng ml^−1^ IL-12 with or without 60 nM FXII for 72 h. Mean clinical and mean cumulative scores±s.e.m. over time of three independent experiments are given (non-parametric Mann–Whitney *U*-test). (**b**) BM chimeras were created by transferring WT and *Cd87*^−/−^ BM into WT and *Cd87*^−/−^ recipient mice after radiation. Mean clinical and mean cumulative scores±s.e.m. of EAE from three independent experiments are shown (non-parametric Mann–Whitney *U*-test). (**c**,**d**) Brain-infiltrating leukocytes (BILs) were separated into cDC^+^ and cDC^−^ by sorting via flow cytometry based on indicated surface markers at *d*_max_ after EAE induction. Both subsets from WT or *F12*^–/–^ mice were analysed for *Il-6* expression by real-time reverse transcription–PCR using 18s rRNA for normalization. Data are given as mean±s.e.m. of two experiments, each experiment generated from pooled brain and spinal-cord-derived cells of *n*=6–7 mice per group and presented as fold change in normalized gene expression relative to WT controls. (**e**) Flow cytometric analysis of BILs from WT and *F12*^−/−^ animals at *d*_max_ after EAE induction determined the expression of CD80, CD86 and MHC-II in cDCs that were pre-gated for CD45^high^CD11b^+^CD11c^+^ cells. Data are representative of two independent experiments with four mice per genotype. (**f**) Histological analysis of spinal cord sections stained for the nucleus (4,6-diamidino-2-phenylindole (DAPI), blue), FXII (red) and CD11c (green) from the lumbar region of EAE WT animals at *d*_max_. Scale bar, 100 μm. *P<0.05.

We obtained consistent results using DC-CD4^+^ T-cell co-cultures. Under unskewed co-culture conditions (WT or *Cd87*^−/−^ CD4^+^ T cells and WT DCs), FXII reduced IFN-γ and increased IL-17A, IL-6 and IL-23 (Fig. 4g). In contrast, cytokine levels of *Cd87*^−/−^ DCs co-cultured with WT or *Cd87*^−/−^ CD4^+^ T cells were not significantly different from controls (Fig. 4g). To prove that CD87 is also relevant *in vivo*, an AT model was created using CD4^+^ T cells and DCs isolated from WT and *Cd87*^−/−^ mice restimulated in the presence or absence of FXII (Fig. 5a). Interestingly, WT mice treated with FXII-restimulated WT CD4^+^ T cells and DCs displayed an aggravated disease course, while FXII had no additional effect on CD87-deficient CD4^+^ T cells and DCs. Of note, transfer of *Cd87*^−/−^ CD4^+^ T cells and DCs led to an attenuated disease course, as compared with control conditions (Fig. 5a).

To address a relevant role of CD87 also on resident cells, bone marrow (BM) chimeras were created by transferring WT or *Cd87*^−/−^ BM into WT and *Cd87*^−/−^ hosts after irradiation. *Cd87*^−/−^ or WT mice reconstituted with *Cd87*^−/−^ BM were less susceptible to the development of EAE compared with WT mice reconstituted with WT BM (Fig. 5b). Interestingly, transfer of WT BM into *Cd87*^−/−^ animals led to a partial protection (Fig. 5b) arguing for a role of FXII on both peripheral and resident cells.

CD87 is also expressed on endothelial, microglial and astroglial cells (Supplementary Fig. 8a). Hence, we isolated MBMECs from WT mice and treated them with FXII under naive and inflammatory conditions. While inflammation led to a significant reduction of transendothelial resistance (TER), FXII had no additional effect (Supplementary Fig. 8b). Furthermore, while MIP-1β production of MBMECs was increased in the presence of FXII, other chemokines were not influenced (unaltered level of CXCL1, CXCL9, CXCL10, CCL2, MIP-1α, RANTES, CCL11, CCL17 and CCL22; no detection of CCL20, CXCL5 and CXCL13; Supplementary Fig. 8c). To exclude a direct effect of FXII on transmigration, we analysed the impact of FXII on the migratory capacity of immune cells in an *in vitro* transmigration assay. The number of immune cells crossing the MBMEC barrier did not change in the absence or presence of FXII (Supplementary Fig. 8d). To analyse the potential effect of FXII on microglia and astrocytes, primary cultures were prepared. However, FXII did not affect major histocompatibility complex (MHC)-I or –II, or production of IL-6, IL-10 and tumour necrosis factor α (TNF-α) cytokines in both microglia (Supplementary Fig. 8e,g) and astrocytes (Supplementary Fig. 8f,h). Of note, the number of activated microglia and astrocytes was unchanged in FXII-deficient animals compared with WT controls on *d*_max_ of EAE (Supplementary Fig. 8i,j).

As extensive FXII depositions were found in the CNS of immunized mice (Fig. 1a,b), we analysed the possible effect of FXII on cDCs present in the CNS on *d*_max_ of EAE mice. Therefore, we separated cDCs from the CNS on *d*_max_ (Fig. 5c). Interestingly, cDCs isolated from *F12*^−/−^ mice showed again a reduction in IL-6 levels (Fig. 5d), while the surface markers CD80, CD86 and MHC-II were unchanged (Fig. 5e). Furthermore, FXII depositions were localized in the close proximity of DCs in the inflamed spinal cord (Fig. 5f).

### Pharmacologic inhibition of FXII protects from EAE

**Figure 6:**
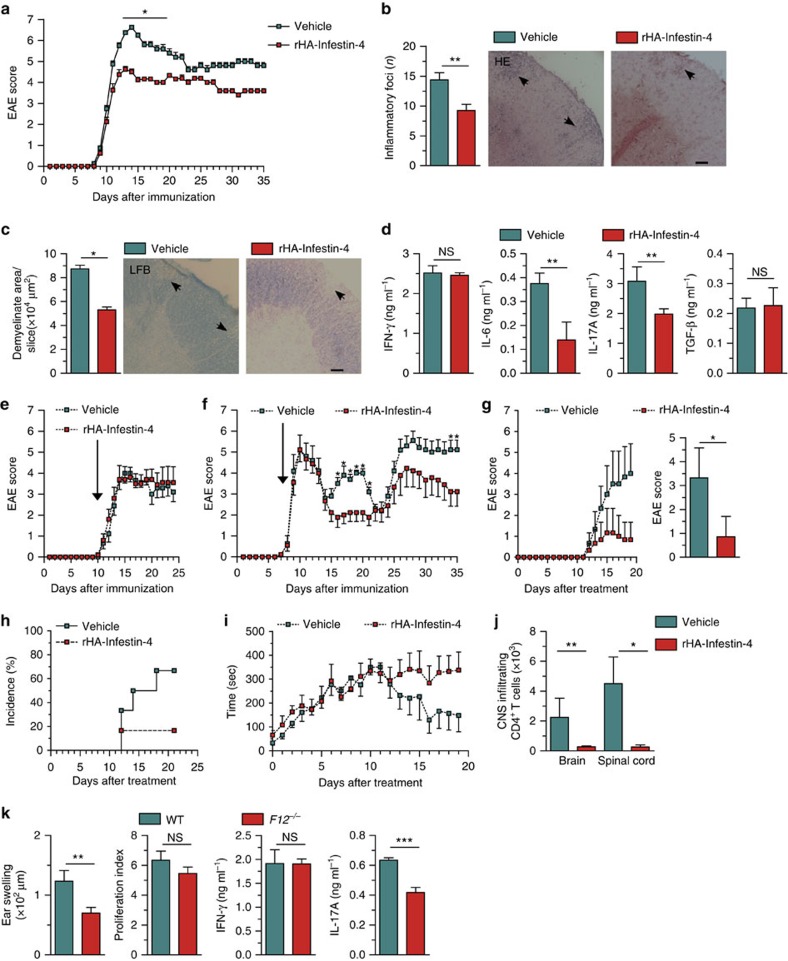
FXII blockade protects from neuroinflammation and contact hypersensitivity. (**a**) Clinical scores of WT mice treated with rHA-Infestin-4 or vehicle starting on day 1 after EAE induction are shown. Representative data from two independent experiments are depicted (Supplementary Table 3). (**b**,**c**) Histological analysis of spinal cord sections of WT and rHA-Infestin-4-treated mice at *d*_max_. Lumbar sections were stained with haematoxylin and eosin (HE) (**b**) to evaluate inflammatory foci or immunostained for Luxol fast blue (LFB) (**c**) to assess demyelination. Arrows indicate inflammation or demyelinated areas. Scale bars, 100 μm. (**d**) Cytokines produced by CD4^+^ T lymphocytes and DCs from WT mice treated with rHA-Infestin-4 or vehicle 10 days after immunization. Data from three independent experiments, each performed in duplicate are shown. Clinical scores of (**e**) MOG_35–55_-immunized WT mice and (**f**) proteolipid protein peptide 139–151-immunized SJL/JRj mice treated with rHA-Infestin-4 or vehicle starting at the first day of neurologic symptoms (arrow; for detailed information, see Supplementary Table 4). (**g**) Clinical, cumulative EAE scores and (**h**) disease incidence of Devic mice treated with rHA-Infestin or vehicle starting at postnatal day 20. (**i**) The motor coordination of Devic mice was assessed using the rotarod. The time the mice remained on the rod was recorded. For each mouse, the average time of three trials followed by 30-min breaks was recorded daily. (**j**) Number of infiltrating CD4^+^ T cells within the central nervous system of Devic mice was analysed by flow cytometry at postnatal day 40. (**k**) Ear swelling of WT or *F12*^−/−^ mice following induction of contact hypersensitivity. Representative data from three independent experiments are shown. Quantification of 2,4-dinitrobenzenesulfonic acid sodium salt-induced proliferation, and IFN-γ and IL-17A production of LN cells from WT and *F12*^−/−^ mice. Representative data in quadruplicate wells from two independent experiments are shown. In **a**–**j**, 200 mg kg^−1^ body weight of rHA-Infestin-4 or vehicle was given once daily. In **a**–**k**, data are given as mean±s.e.m. *P<0.05, **P<0.01, ***P<0.001 (for **a**,**d** and **e**–**k**, non-parametric Mann–Whitney *U*-test; for **b** and **c**, Student's *t*-test); NS, not significant.

Since FXII deficiency prevented EAE, we investigated whether the effect of FXII deficiency could be mimicked using the highly specific FXII inhibitor, recombinant human albumin (rHA)-Infestin-4 (ref. 28). Indeed, rHA-Infestin-4, starting 1 day after MOG_35–55_ immunization, significantly attenuated the signs of EAE in WT mice (Fig. 6a; Supplementary Table 3) and was able to ameliorate cellular inflammation and demyelination (Fig. 6b,c). Again, the protective effect of rHA-Infestin-4 in WT mice was associated with a reduced T_H_17 immune response (Fig. 6d). In contrast, rHA-Infestin-4 could no longer alter the EAE disease course when applied after neurologic symptom onset (Fig. 6e). Interestingly, rHA-Infestin-4 could not be found in the CNS of EAE mice, indicating no significant entrance of the substance through the BBB into the CNS (Supplementary Fig. 9). These findings point again to both a predominant role of FXII in DC-mediated EAE disease priming and influence on resident APCs.

To further demonstrate that FXII inhibition represents a promising new approach to combat MS, we challenged SJL/JRj mice in a relapsing–remitting-EAE (RR-EAE) model^29^. FXII inhibition significantly reduced the number and severity of relapses, even when rHA-Infestin-4 was injected in a delayed setting, that is, not until the first clinical attack (Fig. 6f; Supplementary Table 4). Furthermore, the inhibition of FXII was challenged in a spontaneous model of EAE that has been used in our group before^30^. For this purpose, double transgenic Devic mice were generated by crossbreeding transgenic mice carrying either a MOG_35–55_-specific T-cell receptor (TCR) or MOG_35–55_-specific B-cell receptor, since 50–70% of these mice spontaneously develop EAE-like symptoms without the administration of any substances used for immunization^31^. Littermates were treated daily with rHA-Infestin-4 (200 mg kg^−1^ body weight) or vehicle for 20 consecutive days, starting at postnatal day 20. Devic mice treated with rHA-Infestin-4 displayed attenuated clinical symptoms (Fig. 6g,h), sustained motor coordination, as revealed by rotarod analysis (Fig. 6i), and a limited number of CNS-infiltrating CD4^+^ T cells (Fig. 6j).

To further analyse the functional relevance of FXII in adaptive immunity, we introduced a skin contact hypersensitivity model in which T_H_17 cells are critically involved in disease pathogenesis. In this model, mice are immunized by painting dinitrofluorobenzene on the shaved abdomen on day 0 and undergo antigen rechallenge on day 5 by painting the same antigen on the ear. Ear thickness and the underlying immune responses are assessed 1 day later. *F12*^−/−^ mice developed less inflammation in the challenged ear and less IL-17A production from T cells, while proliferation and IFN-γ production were unchanged (Fig. 6k), suggesting that FXII deficiency can inhibit T_H_17 cell development, not only under neuroinflammatory conditions but also in other immune disorders.

### FXII is upregulated in MS patients

**Figure 7:**
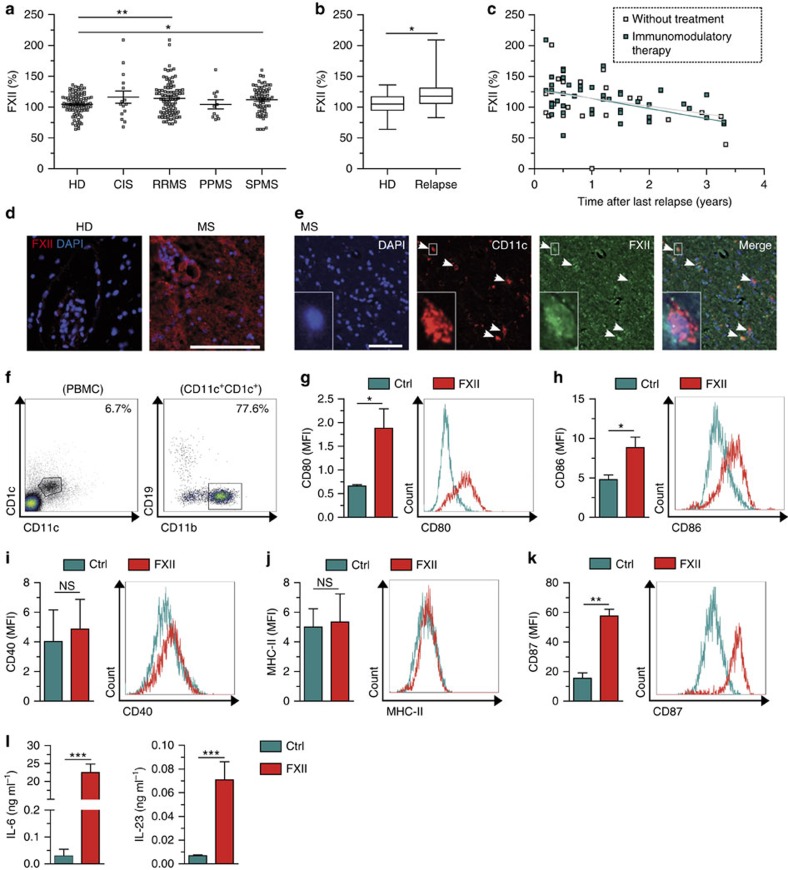
Evidence for the involvement of FXII in human autoimmune CNS inflammation. (**a**) FXII plasma levels in individuals with clinically isolated syndrome (CIS) and MS patients (relapsing–remitting MS (RRMS), primary progressive MS (PPMS) and secondary progressive MS (SPMS)) compared with HDs. (**b**) FXII plasma levels in individuals with RRMS during relapse compared with HDs. (**c**) Correlation of FXII plasma levels with relapse-free time in individuals with RRMS. *R* value: −0.4226. (**d**) Histological analysis of CNS tissue from individuals with MS or from HDs. Sections were stained for FXII (red) and nucleus (4,6-diamidino-2-phenylindole (DAPI), blue). Scale bar, 100 μm. (**e**) Histological analysis of CNS tissue of individuals with MS. Sections were stained for CD11c (red), FXII (green) and nucleus (DAPI, blue). Scale bar, 100 μm. (**f**–**k**) Flow cytometric analysis of human PBMCs from HDs that were incubated with medium only (Ctrl) or stimulated with 60 nM FXII for 24 h. Cells were gated for CD1c^+^CD11c^+^CD11b^+^CD19^neg^ (cDC) based on indicated surface markers (shown in **f**) and their expressions of (**g**) CD80, (**h**) CD86, (**i**) CD40, (**j**) MHC-II and (**k**) CD87 were determined. Representative fluorescence-activated cell sorting plots for indicated surface markers are shown. (**l**) Cytokine concentrations of IL-6 (left panel) and IL-23 (right panel) in the supernatants of human PBMCs treated with 60 nM FXII or untreated (Ctrl) for 24 h. In **a**,**b** and **g**–**l**, data are given as mean±s.e.m. (non-parametric Mann–Whitney *U*-test or Student's *t*-test). *P<0.05, **P<0.01, ***P<0.001; MFI, mean fluorescence intensity; NS, not significant.

To assess the involvement of FXII in humans, we analysed the plasma from individuals with different forms of MS (clinically isolated syndrome, relapsing–remitting MS, primary progressive MS and secondary progressive MS, Supplementary Table 5). FXII plasma level was significantly increased in patients with relapsing–remitting MS and secondary progressive MS compared with healthy donors (HDs; Fig. 7a). Interestingly, enhanced FXII amount was observed during the course of a relapse (Fig. 7b). Moreover, FXII levels tended to correlate with disease activity, as higher levels were associated with a shorter relapse-free period, independent of immunomodulatory therapy (Fig. 7c). In addition to these peripheral alterations, extensive FXII depositions could be found in the CNS of individuals with MS, but not in HDs (Fig. 7d). These depositions were in close contact with DCs, indicating that these cells may be the potential targets of FXII in the CNS of MS patients as well (Fig. 7e). As mouse data suggest an immunomodulatory function of FXII on DCs, also in the periphery, we examined the influence of FXII on these cells in humans (Fig. 7f). Interestingly, in contrast to the mouse data, FXII led to an extensive upregulation of the co-stimulatory molecules CD80 and CD86 in human DCs (Fig. 7g,h), while CD40 and MHC-II expression was unaltered (Fig. 7i,j). Furthermore, CD87, the proposed binding partner of FXII, was significantly increased in human DCs on FXII treatment (Fig. 7k). Together, these data suggest that FXII also significantly enhanced the amount of IL-6 and IL-23 (Fig. 7l), indicating a significant immunomodulatory role of FXII, not only in mice but also in humans.

## Discussion

Immune inflammation triggered by pathogenic and/or autoreactive T_H_ cells underlies various immune diseases^3^,^32^,^33^. To our knowledge, our work represents major novel insights into the role of a plasmatic coagulation cascade member as a critical trigger of autoimmune inflammation. Genetic deficiency or pharmacologic blockade of FXII significantly protected from EAE in different clinically relevant settings. Mechanistically, FXII stimulates DC-mediated T_H_17-cell generation in a CD87-dependent manner. Importantly, FXII deficiency also ameliorated skin allergy, thereby underpinning the pathophysiologic importance of this coagulation factor in adaptive immunity, and thus as a potential therapeutic target in autoimmunity.

The traditional view of plasmatic coagulation cascade is linked to thrombotic diseases^19^. However, there is increasing evidence that some coagulation factors may also be involved in autoimmune disorders, such as in MS, inflammatory bowel disease or dermatitis^34^,^35^,^36^. Depositions of fibrinogen and the degradation products thereof were found in the CNS and skin of patients with MS or dermatitis^34^,^35^,^36^. In the brain, those depositions fostered microglia activation and subsequent plaque formation^12^,^36^. Moreover, a close correlation between the cerebral or spinal fibrin load and the number of relapses was reported in EAE mice, and anticoagulant administration or fibrinogen depletion reversed the pathology^14^,^37^. Apart from triggering thrombus formation via the intrinsic pathway of blood coagulation, FXII is also central to the triggering of the proinflammatory KKS, leading to the formation of BK. The physiologic and pathophysiologic effects of BK are mediated by two distinct BK receptors^38^. Human studies revealed that B1R is expressed on circulating lymphocytes and infiltrating T cells during active episodes of MS. Moreover, B1R can be found on endothelial cells within MS plaques^39^,^40^,^41^. In the EAE model, contradictory reports exist with regard to the relevance of B1R and B2R^21^,^42^.

While the immune system of FXII-deficient mice was dominated by a protective response with a reduction in T_H_17 cells, we found that, for this shift, neither the coagulation system nor the KKS was required. This unexpected observation led us to hypothesize that FXII might affect the immune system via CD87 (ref. 18).

CD87 is a glycoprotein tethered to the cell membrane with a glycosylphosphatidylinositol anchor. CD87 was originally identified as a binding site for urokinase on the surface of different cells, but can also be activated by FXII^18^. Indeed, cDCs and pDCs isolated from the periphery constitutively expressed CD87, as did activated CD4^+^ T cells. Most notably, FXII tipped the balance of T-cell differentiation towards a T_H_17 phenotype, which is increasingly being recognized as a key player in MS pathophysiology^8^,^43^. This effect of FXII critically depended on the presence of CD87 in cDCs. Moreover, since CD87 lacks an intracellular domain, we identified the CD11b integrin, which was previously shown to interact with CD87 on the membrane^24^,^25^, as the adapter-mediating intracellular signalling and cytokine upregulation triggered by FXII.

Our data suggest that FXII acts on peripheral DCs, thereby shaping T-cell differentiation and adaptive immunity. FXII was also found in the CNS during neuroinflammation, indicating a significant local role. Resident cells such as microglia, astrocytes and endothelial cells are known to express CD87. However, none of these cells could be identified as a relevant target of FXII in our model. Nevertheless, FXII was able to induce a cytokine shift in DCs localized in the CNS during neuroinflammation. DCs can regulate T-cell function and are capable of integrating a myriad of incoming immune signals to govern downstream circuits^44^,^45^. We here provide evidence that CD87 activation through FXII impacts the cytokine responses to inflammatory signals and yields a typical T_H_17 cytokine network by boosting IL-6 production under neuroinflammatory conditions.

From a translational perspective, FXII blockade in WT mice with the highly specific FXII inhibitor rHA-Infestin-4 had a beneficial effect on disease severity and progression in different EAE models. Since rHA-Infestin-4 has anticoagulant properties and potentially interferes with hemostasis, there might be a considerable risk of inducing bleeding complications when using this compound. However, previous experimental stroke and thrombosis studies revealed that neither *F12*^−/−^ nor WT mice treated with rHA-infestin-4 developed a bleeding phenotype^19^,^28^. This is in line with the notion that FXII is dispensable for hemostasis, but only contributes to thrombosis^16^. The fact that FXII inactivation does not affect hemostasis makes FXII inhibition a safe pharmacologic approach, at least in rodents^46^. Whether this also applies to humans, and particularly in the course of autoimmune CNS inflammation, needs to be elucidated. Notably, we never observed any major haemorrhages in the organs of *F12*^−/−^ mice subjected to EAE (not shown).

Overall, our study identifies FXII as a key regulator of adaptive immune responses during neuroinflammation. Targeted inhibition of FXII might become a promising approach to redress immune balance in MS. Further studies in relevant preclinical disease models are warranted.

## Methods

### Mice

C57BL/6N mice, which served as controls (WT), and SJL/JRj mice were purchased from Charles River Laboratories (Sulzfeld, Germany). FXII-deficient (*F12*^−/−^)^19^, factor XI-deficient (*F11*^−/−^)^19^, B1R-deficient (*B1r*^−/−^)^21^ and B2R-deficient (*B2r*^−/−^) mice^21^ were a gift from C. Kleinschnitz (Department of Neurology, University Hospital Würzburg, Würzburg, Germany). CD11b-deficient (*Itgam*^−/−^)^47^ mice and CD87-deficient (*Cd87*^−/−^)^48^ animals were obtained from T. Chavakis (Department of Clinical Pathobiochemistry and Institute for Clinical Chemistry and Laboratory Medicine, University Clinic Carl Gustav Carus, Technische Universität of Dresden, Dresden, Germany) and C. Ruppert (Department of Internal Medicine, Universities of Giessen & Marburg Lung Center (UGMLC)/Member of the German Center for Lung Research (DZL), Justus-Liebig University, Giessen, Germany), respectively. Transgenic 2D2 (*TCR*^MOG^) and Th (*IgH*^MOG^) mice from the laboratory of H. Wekerle (Department of Neuroimmunology, Max Planck Institute for Neurobiology, Martinsried, Germany) were also used^31^. All mice, except SJL/JRj, had a C57BL/6 background. All animal experiments were performed on adult mice at 8–10 weeks of age. All animal protocols were approved by the local authorities, and were in accordance with the German laws and regulations for animal care.

### Induction and evaluation of EAE

Active EAE was induced by immunization of 8–10-week-old female *F12*^−/−^, *F11*^−/−^ and WT (C57BL/6N) mice with MOG_35–55_ peptide (Charité, Berlin, Germany)^21^. Briefly, animals were subcutaneously immunized with 200 μg of the mouse MOG_35–55_ peptide emulsified in 200 μl complete Freund's adjuvant (Sigma-Aldrich Chemie GmbH, Steinheim, Germany) containing 200 μg *Mycobacterium tuberculosis* (strain H37 Ra; Becton, Dickinson and Company (BD), Sparks, MD, USA). Pertussis toxin (PTx; 400 ng in 200 μl PBS; Enzo Life Sciences, Farmingdale, NY, USA) was injected intraperitoneally (i.p.) on the day of immunization (day 0) and 2 days later. Some mice were treated daily with intravenous (i.v.) injections of FXII (200 mg kg^−1^ body weight, Haematologic Technologies, Essex Junction, VT, USA) or respective vehicle from MOG_35–55_ immunization. Furthermore, FXII inhibitor rHA-Infestin-4 (200 mg kg^−1^ body weight; CSL Behring, Marburg, Germany) or respective vehicle were administered by daily i.v. injection starting 1 day after MOG_35–55_ immunization (prophylactic regimen) or 1 day after the appearance of first clinical signs (therapeutic regimen). For AT-EAE experiments^49^,^50^, WT, *F12*^−/−^ or *Cd87*^−/−^ donor mice were immunized as described above for active EAE experiments, but animals received a dose of 200 ng PTx on the day of immunization and 2 days later. LN cells were isolated 12 days post immunization, and, in some cases, further separated into CD4^+^ T cells as well as CD11c^+^ APCs as described below. LN cells or CD4^+^ T and CD11c^+^ cells (ratio 5:1, conditions as described in the figures) were restimulated for 72 h with 10 μg ml^−1^ MOG_35–55_ peptide and 0.5 ng ml^−1^ IL-12 (R&D systems, Minneapolis, MN, USA). AT of the encephalitogenic LN cells (8.4 × 10^6^ cells per mouse) or CD4^+^ T and CD11c^+^ cells (1.5 × 10^7^ cells per mouse) in 200 μl PBS was performed by i.p. injection into recipient mice. For the generation of BM chimera^51^, WT and *Cd87*^−/−^ mice were irradiated with a single dose of 10 Gy, and BM cells from WT or *Cd87*^−/−^ mice from the same litter were injected i.v. into the irradiated mice (4.0 × 10^6^ cells per mouse). Six weeks later, active EAE was induced as above described. RR-EAE was induced in SJL/JRj mice by vaccination with 75 μg of proteolipid protein peptide 139–151 (Genemed Synthesis, San Antonia, TX, USA) emulsified in complete Freund's adjuvant containing 600 μg *M. tuberculosis*^29^. Each mouse received subcutaneous injections of 200 μl emulsion divided among four sites draining axillary and inguinal LN. PTx (400 ng) was administered i.p. on the day of immunization and 2 days later. Animals were scored as described below. rHA-Infestin-4 (200 mg kg^−1^ body weight) or respective vehicle was administered daily i.v. after the onset of the first clinical RR-EAE attack. A relapse was defined as a sustained (>2 day) increase in the clinical score by at least one full grade after the animal had improved previously by at least one full grade and had stabilized for at least 2 days. For a spontaneous model of EAE, 2D2 mice were crossed with Th mice (*IgH*^MOG^) to generate double transgenic Devic mice (*TCR*^MOG^ × *IgH*^MOG^) in our facility^30^. Littermates received daily i.p. injections of rHA-Infestin-4 (200 mg kg^−1^ body weight) or vehicle for 20 consecutive days, starting at postnatal day 20. The motor coordination was assessed using the rotarod test. For this, mice were placed on a rotarod with automatic timers and falling sensors (Harvard Apparatus March-Hugstetten, Germany) while the speed of the rod was accelerated from 4 to 40 r.p.m. within 10 min, and the time the mice remained on the rod was recorded. For each mouse, the average time of three trials followed by 30-min breaks was recorded daily. All animals were kept under standard conditions and had access to water and food *ad libitum*. The clinical course of EAE was monitored daily by two blinded investigators using the following score system: grade 0, no abnormality; grade 1, limp tail tip; grade 2, limp tail; grade 3, moderate hindlimb weakness; grade 4, complete hindlimb weakness; grade 5, mild paraparesis; grade 6, paraparesis; grade 7, heavy paraparesis or paraplegia; grade 8, tetraparesis; grade 9, quadriplegia or premoribund state; or grade 10, death. Animals with a score >7 were killed and the last score observed was included in the analysis until the end of the experiment. The mean cumulative score for a treatment group was calculated as the sum of the daily scores of all animals from day 0 until the end of the experiment divided by the number of animals in the respective group.

### Cell preparation

Single-cell suspensions of mouse LN (cervical, axillary, mesenterial and inguinal), spleens or BM were prepared from naive animals, or immunized animals 10 days after the induction of EAE or at the peak of disease^21^. CD4^+^ T cells were isolated from LN cells of WT, *F12*^−/−^ and *F11*^−/−^ mice using CD4^+^ or CD4^+^CD62L^+^ T-cell isolation kits (Miltenyi Biotec, Bergisch Gladbach, Germany). DCs were purified from the spleen or BM of WT, *F12*^−/−^, *Cd87*^−/−^ and *Itgam*^−/−^ mice using the CD11c^+^ or pDC isolation kits (Miltenyi Biotec). All immunomagnetic cell separations were performed according to the supplier's manual. For purification of BILs^52^, brains and spinal cords from immunized WT and *F12*^−/−^ animals at *d*_max_ were collected after transcardial PBS perfusion. CNS tissues were cut into pieces and further mechanically homogenized in PBS, layered on a 30–50% Percoll (Sigma-Aldrich) gradient and continuously centrifuged for 30 min at 2,500 r.p.m. Mononuclear cells were isolated at the interphase. After isolation, cells were washed and resuspended in the appropriate buffer or medium for further analysis. For quantification of cell numbers isolated from the CNS, Calibrite beads (BD) were added to freshly isolated BILs from WT and *F12*^−/−^ animals before washing and staining. Furthermore, human PBMCs were obtained from leukoreduction system chambers (Department of Transfusion Medicine, University of Münster) of anonymous HDs by density gradient centrifugation as described before^53^. Purified PBMCs were left untreated or treated with 60 nM FXII for 24 h before the use for further analysis by enzyme-linked immunosorbent assay (ELISA) and flow cytometry. For rRT–PCR, CNS-derived DC subsets were isolated from the spinal cord and brain of immunized WT and *F12*^−/−^ animals at *d*_max_ by sorting on a BD fluorescence-activated cell sorting Aria III instrument after BIL purification. Cell purity was routinely >95%.

### ELISA and proliferation assay

Samples of serum, plasma, single-cell suspensions of LN and CSF were taken from mice under basal conditions or at *d*_max_ of EAE from respective mice. CSF was collected according to the technique described by Fleming *et al.*^54^ Samples (5 μl) were diluted 20 times in 50 mM Tris-HCl buffer, pH 7.4, containing 100 mM NaCl and 0.05% Tween-20, and frozen at −80 °C. Determination of FXII was performed using the ELISA Kit for Coagulation FXII (USCN Life Science, Inc., Wuhan, China) according to the manufacturer's instructions. In one set of experiments, irradiated (35 Gy) DCs from WT or *F12*^−/−^ mice were pulsed with 10 μg ml^−1^ MOG_35–55_ and used as APC co-cultured with syngeneic CD4^+^ T cells derived from the LN, as described before^52^. Cells were cultured in 1 ml DMEM containing 10 mM HEPES, 25 μg ml^−1^ gentamicin, 50 μM mercaptoethanol, 5% fetal calf serum (FCS), 2 mM glutamine and 1% non-essential amino acids (Cambrex, Verviers, Belgium) for 2 days and stimulated with 10 μg ml^−1^ MOG_35–55_. [^3^H]thymidine (Amerham, Piscataway, NJ, USA) was added for the final 14 h, and radioactivity was measured on a β-scintillation counter (TopCount NXT, PerkinElmer, Rodgau-Jügesheim, Germany). The supernatants were analysed for different cytokines by ELISA. Fractioned DC subpopulations were incubated for 48 h with medium alone, 1 μg ml^−1^ LPS (for cDCs) or 10 μg ml^−1^ CpG oligodeoxynucleotide 1,826 (for pDCs) in the absence or presence of FXII, non-cleavable FXII (CSL Behring) or FXIIa (American Diagnostica Inc.—now Sekisui Diagnostics, Stamford, CT, USA). Cytokine production from BILs was measured in culture supernatants collected after a 24-h cell incubation with 10 μg ml^−1^ MOG_35–55_. Mouse cytokines (IL-6, IL-10, IL-12, IL-17A, IL-23, IL-27, IFN-γ and the active form of TGF-β1) and human cytokines (IL-6 and IL-23) were assessed in culture supernatants of the above-described cells by ELISA using specific kits (R&D Systems, Peprotech, Hamburg, Germany, and eBioscience, San Diego, CA, USA) according to the manufacturer's instructions. The concentrations of cAMP in cDCs were measured using cAMP XP assay kit (Cell Signaling Technology, Cambridge, UK). Briefly, cDCs were incubated with medium only or with 1 μg ml^−1^ LPS for 10 min in the absence or presence of FXII. Reactions were stopped by aspirating the medium, and then the cells were lysed with 100 μl of lysis buffer. Cell lysate was transferred into microtiter plates, and cAMP concentrations were measured according to the manufacturer's protocol. BK levels were determined using the Bradykinin ELISA Kit (Phoenix Pharmaceuticals, Inc., Karlsruhe, Germany) with minor modifications. Briefly, biotin-labelled antigen was diluted 20 times to increase the sensitivity. The competitive immunoextraction step was carried out by incubating for 4 h at room temperature (RT) under agitation. Peptides were detectable at a 1–10 pg ml^−1^ linear range with 0.1 pg ml^−1^ minimum detectable concentration^42^. For complement (C5a) detection, plates were precoated with a rat anti-mouse C5a capture antibody (clone I52-1486; BD) diluted in coating buffer (100 μM NaHCO_3_ and 34 μM Na_2_CO_3_, pH 9.5), and incubated overnight^55^. This capture antibody is specific for a neoepitope exposed only in mouse C5a/C5adesArg, and does not cross react with native C5. Then, plates were blocked for 1 h in assay diluent (10% FCS/PBS, followed by a 2-h incubation with samples and standards. Thereafter, a rat anti-mouse C5a biotin-labelled detection antibody (clone I52-278; BD) was added for 1 h. To detect captured mouse C5a, streptavidin–horseradish peroxidase (BD) was added for 30 min, followed by tetramethylbenzidine (Sigma-Aldrich); the reaction was stopped using 1 M H_2_SO_4_, and the plate was read at 450 nm. Concentrations of measured analytes were calculated via four-parameter logistic curve fitting according to standard curves, respectively. For *in vitro* cell treatments, FXII, non-cleavable FXII and FXIIa were diluted in medium to a final concentration of 60 nM, unless otherwise specified.

### Histology and immunohistochemistry/-cytochemistry

For murine CNS tissue, spinal cords were removed after transcardial perfusion with PBS from naive WT mice or from MOG_35–55_-immunized WT, *F11*^−/−^ and *F12*^−/−^ mice at *d*_max_, embedded in Tissue-Tek optimum cutting temperature (OCT) compound (Miles Laboratories, Elkhart, IN, USA), and cut into 10-μm-thin sections from the lumbar region. For human CNS tissue, human autopsy and biopsy material from MS patients and HDs was obtained from The UK Multiple Sclerosis Tissue Bank (Division of Neuroscience and Mental Health, London, UK, *n*=5). For the present study, five MS cases were analysed for whom cryofixed brain tissue was available. The lesions fulfilled the morphologic criteria of an inflammatory demyelinating process consistent with MS when stained with haematoxylin and eosin (HE), Luxol fast blue (LFB)-periodic acid Schiff myelin stain and Bielschowsky's silver impregnation for axons. Cryofixed tissues from MS patients and HDs were cut into 10-μm sections. For immunohistochemistry/-cytochemistry, sections or cell cultures were postfixed with 4% paraformaldehyde for 10 min at RT, washed with 10 mM PBS, blocked for 1 h at RT with 1 × PBS containing 5% bovine serum albumin (BSA), 1% normal goat serum and 0.3% Triton X-100 (Sigma), and incubated with primary antibody at 4 °C overnight. Primary antibodies were diluted in 1 × PBS containing 5% BSA and 1% normal goat serum, and incubated overnight at 4 °C. Sections were stained with the primary antibodies to the following: FXII (1:50, Proteintech), human CD11c (1:100, clone 3.9, Abcam, Cambridge, UK), human albumin (1:50, Origene Technologies, Rockville, MD, USA), ionized calcium-binding adapter molecule 1 (1:250, polyclonal, Wako Chemicals GmbH, Neuss, Germany), glial fibrillary acidic protein (GFAP; 1:1,000, clone G-A-5, Sigma-Aldrich) and mouse CD11c (1:1,000, clone N418, eBioscience, San Diego, CA, USA). Astrocyte and microglia cultures were stained with anti-GFAP and anti-CD11b (1:1,000, clone EPR1344, Abcam), respectively. In addition, both were stained with anti-MHC-I (1:200, clone ER-HR52, Abcam) and anti-MHC-II (1:50, clone NIMR-4, Abcam). After washing steps, secondary antibodies were diluted in 1 × PBS containing 1% BSA and incubated for 1 h at RT. The following secondary antibodies were used: Alexa Fluor 488-coupled goat anti-mouse (1:100, polyclonal, Invitrogen, Waltham, MA USA) or goat anti-Armenian hamster (1:100, polyclonal, Invitrogen), and Cy3-coupled goat anti-rabbit or anti-mouse (1:800, polyclonal, Dianova, Hamburg, Germany). All stainings were embedded in peqGOLD (Peqlab, Erlangen, Germany) before microscopic analysis. Negative controls were obtained by either omitting the primary or secondary antibody and revealed no detectable signal (not shown). To reveal inflammatory infiltrates and demyelination^21^, sections were stained with HE and LFB according to the standard procedures. All stainings were examined by microscopy (Axiophot2, Zeiss, Oberkochen, Germany) with a charge-coupled device camera (Zeiss, Oberkochen, Germany) and analysed in a blinded manner using Axiovision (Zeiss) and also Image J software. For quantification, inflammatory foci (HE), demyelinated areas (LFB) and the fluorescence intensity of ionized calcium-binding adapter molecule 1 and GFAP were counted on three slices of six mice per group, and the fluorescence intensity of MHC-I and MHC-II per area was calculated for five coverslips per condition, whereas the value of each coverslip is the mean of five cells per coverslip.

### Flow cytometry

For the detection of cell surface markers and intracellular/intranuclear markers by flow-activated cell-sorting analysis, single-cell suspensions from murine LN, spleen and BIL of WT and *F12*^−/−^ mice under basal conditions, or 10 days after the induction of EAE or at the peak of disease, as well as fractioned cell populations and human PBMCs were stained for 30 min at 4 °C, with appropriate combination of indicated fluorescence-labelled monoclonal antibodies in PBS containing 0.1% NaN_3_ and 0.1% BSA. Corresponding isotype controls were used for all stainings. For blocking of Fc receptors, cells were preincubated with purified anti-CD16/CD32 antibody (BioLegend, London, UK, 1.0 μg per 10^6^ cells in 100 μl volume) or with human Fc receptor-blocking reagent (Miltenyi Biotec, Bergisch Gladbach, Germany) for 5 min on ice before immunostaining. The following monoclonal antibodies were used at 1:200 dilutions for the detection of cell surface markers: CD3 (clone 17A2, BioLegend), CD4 (clone RM4-5, BioLegend), CD8a (clone 53-6.7, BioLegend), CD11b (clone M1/70, eBioscience), CD11c (clone N418, BioLegend), CD25 (clone PC61, BioLegend), CD40 (clone 1C10, eBioscience), CD45R/B220 (clone RA3-6B2, BD), CD80 (clone 16-10A1, BD), CD86 (clone GL1, BD), CD154 (also known as CD40L, clone MR1, BioLegend), CD317 (clone 927, BioLegend), Ly-6C (clone HK1.4, BioLegend), MHC-II (clone M5/114.15.2, eBioscience) and TCR-γ/δ (clone GL3, BioLegend) for mouse cells; and CD1c (clone AD5-8E7, Miltenyi Biotec), CD11b (clone M1/70, BioLegend), CD11c (clone 3.9, eBioscience), CD19 (clone HIB19, BD), CD40 (clone 5C3, BioLegend), CD80 (clone L307.4, BD), CD86 (clone 2331, BD), CD87 (clone VIM5, eBioscience) and MHC-II (clone G46-6, BD) for human cells. The biotinylated polyclonal anti-mouse CD87 antibody (1:20, R&D Systems) was detected by a following fluorescence-labelled streptavidin-conjugated antibody (1:100, Invitrogen). For immunofluorescent staining of intracellular cytokines, peripheral LN cells as well as CNS infiltrates were isolated 10 days after the induction of EAE or at the peak of disease and polyclonally restimulated for 4 h at 37 °C in the presence of phorbol 12-myristate 13-acetate/ionomycin and brefeldin A, using Leukocyte Activation Cocktail (BD) to block cytokine secretion. In the case of *in vitro* T_H_1 and T_H_17 differentiation (which is described below), cells were treated with brefeldin A (BD) for 8 h after culturing. After cell-specific surface staining, mouse IL-17A (1:50, clone eBio17B7, eBioscience) or IFN-γ (1:50, clone XMG1.2, BD) protein expression was evaluated by intracellular staining using the Cytofix/Cytoperm Plus Kit (BD) according to the manufacturer's protocol. For intranuclear Foxp3 (1:100, clone FJK-16S, eBioscience) analyses, after cell-specific surface staining, cells were fixed/permeabilized with the Foxp3/Transcription Factor Staining Buffer Set (eBioscience) according to the manufacturer's protocol. Stained cells were assayed on a FACSGallios flow cytometer using Kaluza software (Beckman Coulter, Krefeld, Germany).

### rRT–PCR

RNA isolation and RT–PCR were performed as previously described following TaqMan gene expression assays (Applied Biosystems, Foster City, CA, USA)^21^: *Tbx21* (Mm00450960_m1), *Gata3* (Mm00484683_m1), *Rorc* (Mm01261622_m1), *Foxp3* (Mm00475162_m1), *Brdkb1* (B1R, Mm00432059_s1), *Brdkb2* (B2R, Mm00437788_s1), *Cd87* (Mm00440911_m1), *Par1* (Mm00438851_m1), *Par2* (Mm00433160_m1), *Par3* (Mm00473929_m1), *Par4* (Mm01228147_m1), *Il-6* (Mm00446190_m1) and eukaryotic 18S ribosomal RNA (Hs99999901_s1). Results were analysed using the StepOne software (Applied Biosystems) and the comparative Ct (Threshold cycle) method. Data are expressed as 2^−ΔΔCt^ for the experimental gene of interest normalized to the housekeeping gene and presented as fold change relative to control.

### Western blot

Spinal cord was homogenized in radioimmunoprecipitation assay buffer (25 mM Tris (pH 7.4), 150 mM NaCl, 1% NP-40) containing 0.1% SDS and 4% proteinase inhibitor (complete protease inhibitor cocktail (Roche, Basel, Swiss))^19^,^55^. Samples were sonified for 10 s. Afterwards, tissue lysates were centrifuged at 15,000*g* for 30 min at 4 °C, and the supernatants were used for bicinchoninic acid protein assay and subsequent western blot analysis. The total lysates were treated with 4 × SDS–PAGE loading buffer (final concentration: 62.5 mM Tris (pH 6.8), 3% β-mercaptoethanol, 8% SDS, 15% glycerol) at 95 °C for 5 min. A 20-μg amount of total protein was electrophoresed and transferred to a polyvinylidene difluoride membrane. After blocking for 30 min with blocking buffer (5% non-fat dry milk, 50 mM Tris-HCl pH 7.5, 0.05% Tween-20), membranes were incubated with anti-fibrin/fibrinogen polyclonal antibody 1:500 (cross-reactive for fibrin and fibrinogen; Acris Antibodies, Herford, Germany). After a washing step with TBST (50 mM Tris-HCl (pH 7.5), 0.05% Tween-20), membranes were incubated for 1 h with horseradish peroxidase-conjugated donkey anti-rabbit immunoglobulin G (for fibrin/fibrinogen; Dianova) at a dilution of 1:5,000 and were finally developed using ECL plus (GE Healthcare, Hamburg, Germany). Images have been cropped for presentation. Full-size images are presented in Supplementary Fig. 10.

### MBMECs and transmigration assays

MBMECs^21^ were prepared from brains of WT and *B1r*^−/−^ mice, and cultured 6 days before reseeding on Collagen IV/fibronectin- (Sigma) coated Transwell inserts with 3.0 μm pore polyester membrane (Corning, Lowell, MA, USA). Purity, confluency and cell morphology were checked on a regular basis by flow cytometry, resistance measurements and microscopy. To induce upregulation of B1R, MBMECs were inflamed *in vitro* using IFN-γ and TNF-α (each 500 IU ml^−1^, Peprotech) for 24 h. Naive LN cells from WT and *F12*^−/−^ mice were loaded in the upper chamber and were allowed to migrate towards 5% FCS. After an incubation period of 18 h, migrated cells from the lower chamber of two compartments were collected, and the relative cell number of CD4^+^ T cells was determined by flow cytometry. To assess the effects of FXII on MBMECs, the cells were treated with FXII (60 nM) under naive or inflamed conditions with IFN-γ and TNF-α (each 500 IU ml^−1^) for 24 h, during which the transendothelial resistance was monitored with the cellZscope apparatus (nanoAnalytics GmbH, Münster, Germany). Twenty-four hours post inflammation, MBMEC supernatants were collected, and the concentration of proinflammatory chemokines was assessed using the LEGENDplex Mouse Proinflammatory Chemokine immunoassay (catalogue no. 740007; BioLegend) according to the manufacturer's instructions. To assess MBMEC permeability, 24 h post inflammation with or without FXII, naive splenocytes from WT mice were loaded on the upper chamber of the transwell and were allowed to migrate for 18 h towards 5% FCS. For quantification of migrated cells, Calibrite beads were added before collecting the cells from the lower chamber. Numbers of migrated cells were determined by counting 1.0 × 10^4^ references beads by flow cytometry as described before^56^.

### *In vitro* cell differentiation

Purified naive CD4^+^CD62L^+^ T cells were activated for 3 days with 5 μg ml^−1^ of plate-bound CD3-specific (clone 145-2C11; eBioscience) and 1 μg ml^−1^ soluble CD28-specific (clone 37.51; BD) antibodies in the absence or presence of 60 nM FXII, and in the presence of various combinations of recombinant cytokines and blocking antibodies as follows: 10 μg ml^−1^ anti-IL-4 (clone 11B11, eBioscience) and 10 ng ml^−1^ IL-12 (Peprotech) for T_H_1 cells; 5 ng ml^−1^ human TGF-β (R&D Systems), 20 ng ml^−1^ IL-6 (eBioscience), 10 μg ml^−1^ anti-IFN-γ (clone XMG1.2, eBioscience) and 10 μg ml^−1^ anti-IL-4 for T_H_17 cells; 5 ng ml^−1^ TGF-β, 10 μg ml^−1^ anti-IFN-γ and 10 μg ml^−1^ anti-IL-4 for iTreg cells. After 3 days at 37 °C and 5% CO_2_, cells of T_H_1 and T_H_17 differentiation were further analysed by flow cytometry as described above.

### Contact hypersensitivity

We sensitized female mice by applying 25 μl of 1% (w/v) dinitrofluorobenzene in acetone/olive oil (4:1, v/v) on the shaved abdominal skin. After 5 days, animals were rechallenged by applying 20 μl of 0.3% dinitrofluorobenzene to the left ear. To determine ear swelling, ear thickness was measured with a micrometre before and 24 h after antigen re-exposure. LN cells from WT and *F12*^−/−^ mice were prepared and cultured on day 5.

### Primary murine cell cultures

For microglia culture, pups of WT mice were decapitated at postnatal days 1–5 and, after removal of the cerebellum, the meninges were removed. Up to five brains were mechanically homogenized together in 5 ml L-glutamine-containing DMEM supplemented with 10% heat-inactivated FCS, 1% non-essential amino acids and 1% penicillin/streptomycin with a 5-ml pipette. The supernatants were incubated for 5 min on ice, and after centrifugation of the pooled supernatants at 486*g* for 5 min at RT, the pellets were resuspended in 1 ml medium/2–3 brains. The cell suspensions were transferred to poly-L-lysin-precoated 75-cm^2^ cell culture flasks in a total volume of 10 ml each and incubated at 37 °C and 5% CO_2_. After 1 and 7 days of culturing, the medium was changed. After 7 additional days of culturing, microglial cells were selectively detached by carefully knocking against the flask. Suspended microglia cells were pelleted at 300*g* for 5 min at RT, before 60,000 cells in 250 μl medium were seeded for further analysis. For immunocytochemistry and rRT–PCR experiments, cells were seeded on poly-L-lysin-precoated coverslips and cytokine production in the supernatants was assessed by ELISA as described above after treating the cells. Therefore, cells were incubated for 24 h at 37 °C and 5% CO_2_ after seeding to rest, and stimulated with LPS (1 μg ml^−1^) or LPS (1 μg ml^−1^) and FXII (60 nM), or not stimulated for 1 additional day. Astrocyte cell cultures were obtained from WT mice on postnatal days 1–3 as described^51^. In brief, mice were killed by decapitation, their brains were transferred to Hanks' Balanced Salt Solution and the meninges were removed. After preparation of the cortex, tissue was collected in 13.5 ml Hanks' Balanced Salt Solution and incubated for 15 min at 37 °C with 1.5 ml DNAse I and 1.5 ml trypsin 2.5%. Cells were mechanically dissociated and filtered through a 45-μm cell strainer. Then, 4.5 × 10^6^ astrocytes were cultured in 75-cm^2^ flasks in astrocytes medium (1 × minimum essential medium, 1 mM sodium pyruvate, 33.3 mM glucose, 10% horse serum and 1% penicillin/streptomycin). The medium was changed 24 h later and twice per week thereafter. When reaching a confluent layer, astrocytes were trypsinized with 0.25% trypsin and EDTA, and frozen in freezing medium (astrocyte medium and 10% dimethyl sulfoxide). Cells were defrosted and cultured on poly-D-lysine-coated coverslips for 2 weeks in astrocyte medium at 37 °C and 5% CO_2_ for further analysis as described for microglia.

### Patients

Fresh blood samples were obtained from 260 MS patients referred to the Departments of Neurology at the University Hospital Würzburg and the University of Münster. MS diagnosis was made according to the revised criteria of McDonald *et al.*^57^ Of the patients included in the study, 87 had not received immunomodulatory treatment except for corticosteroids, with the last dose administered at least 3 months before study entry. In addition, we included 173 patients who had received different immunomodulatory therapies (glatiramer acetate, interferons, fingolimod, natalizumab, teriflunomide and mitoxantrone). All patients gave informed consent in accordance with the Declaration of Helsinki and a protocol approved by the Ethics Committees of the Universities of Würzburg and Münster. Blood (10–20 ml) was collected by venous puncture using a tourniquet. In parallel, 130 sex- and age-matched HDs were included in the study. FXII activity of plasma samples was determined with an automated blood coagulation system (Siemens Healthcare, Eschborn, Germany) according to the manufacturer's instructions. The percentage of FXII activity was calculated in comparison with standard human plasma and FXII-deficient plasma (Siemens Healthcare, Eschborn, Germany).

### Statistical methods

EAE data were analysed by the non-parametric Mann–Whitney *U*-test. To compare the mean day of onset and maximal score, the Mann–Whitney rank-sum test was used. Paired data were evaluated by the Student's *t*-test. Mann–Whitney *U*-test for parametric data without normality data sets was used. In the case of multiple comparisons, one-way analysis of variance (ANOVA) followed by *post hoc* analysis using Tukey's multiple comparisons test or Kruskal–Wallis test with Dunn *post hoc* analysis was used. Data were analysed using Prism 5.04 (Graph Pad, USA), and the values of probability (*P*)<0.05 were considered as statistically significant. The level of significance was labelled as NS (not significant), *P<0.05, **P<0.01 or ***P<0.001. *In vitro* and *in vivo* data are expressed as mean±s.e.m.)from at least three independent experiments, unless otherwise indicated. The number of animals (*n*=12) necessary to detect a standardized effect size on EAE score ≥0.5 (WT mice versus *F12*^−/−^ mice) was calculated via *a priori* sample size analysis with the following assumptions: *α*=0.05, *β*=0.2 (power 80%), mean and s.d. 20% of the mean (StatMate 2.0; Prism 5.03, Graph Pad). To compare FXII activity in patients, analysis of variance was used.

### Data availability

The authors declare that the data supporting the findings of the study are available within the article and its Supplementary Information.

## Additional information

**How to cite this article:** Göbel, K. *et al.* Blood coagulation factor XII drives adaptive immunity during neuroinflammation via CD87-mediated modulation of dendritic cells. *Nat. Commun.* 7:11626 doi: 10.1038/ncomms11626 (2016).

## Supplementary Material

Supplementary Information Supplementary Figures 1-10 and Supplementary Tables 1-5
