# Supplementary material for: Blood coagulation factor XII drives adaptive immunity during neuroinflammation via CD87-mediated modulation of dendritic cells
Source: Nat Commun. 2016 May 18;7:11626. doi: 10.1038/ncomms11626 (PMC4873982; doi:10.1038/ncomms11626)
Supplement: Supplementary Information — Supplementary Figures 1-10 and Supplementary Tables 1-5 [file ncomms11626-s1.pdf]

Supplementary information

Göbel K., Pankratz S. et al.

Blood coagulation factor XII drives adaptive immunity during neuroinflammation via CD87-mediated modulation of dendritic cells

Supplementary Figure 1

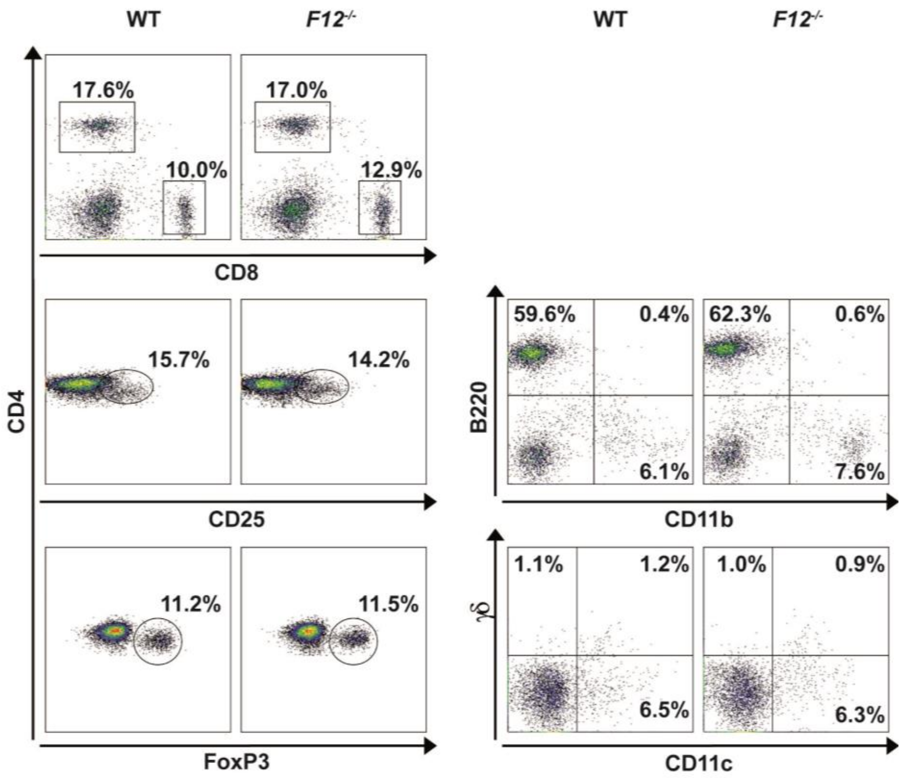

Supplementary Figure 1. Percentages of immune cells are distributed normally in the spleens of naïve *F12*$^{-/-}$ mice compared with controls. Total splenocytes of naïve WT and *F12*$^{-/-}$ animals were analyzed for surface or intranuclear expression of the indicated markers by flow cytometry. Data are representative of three independent experiments with three mice per genotype.

Supplementary Figure 2

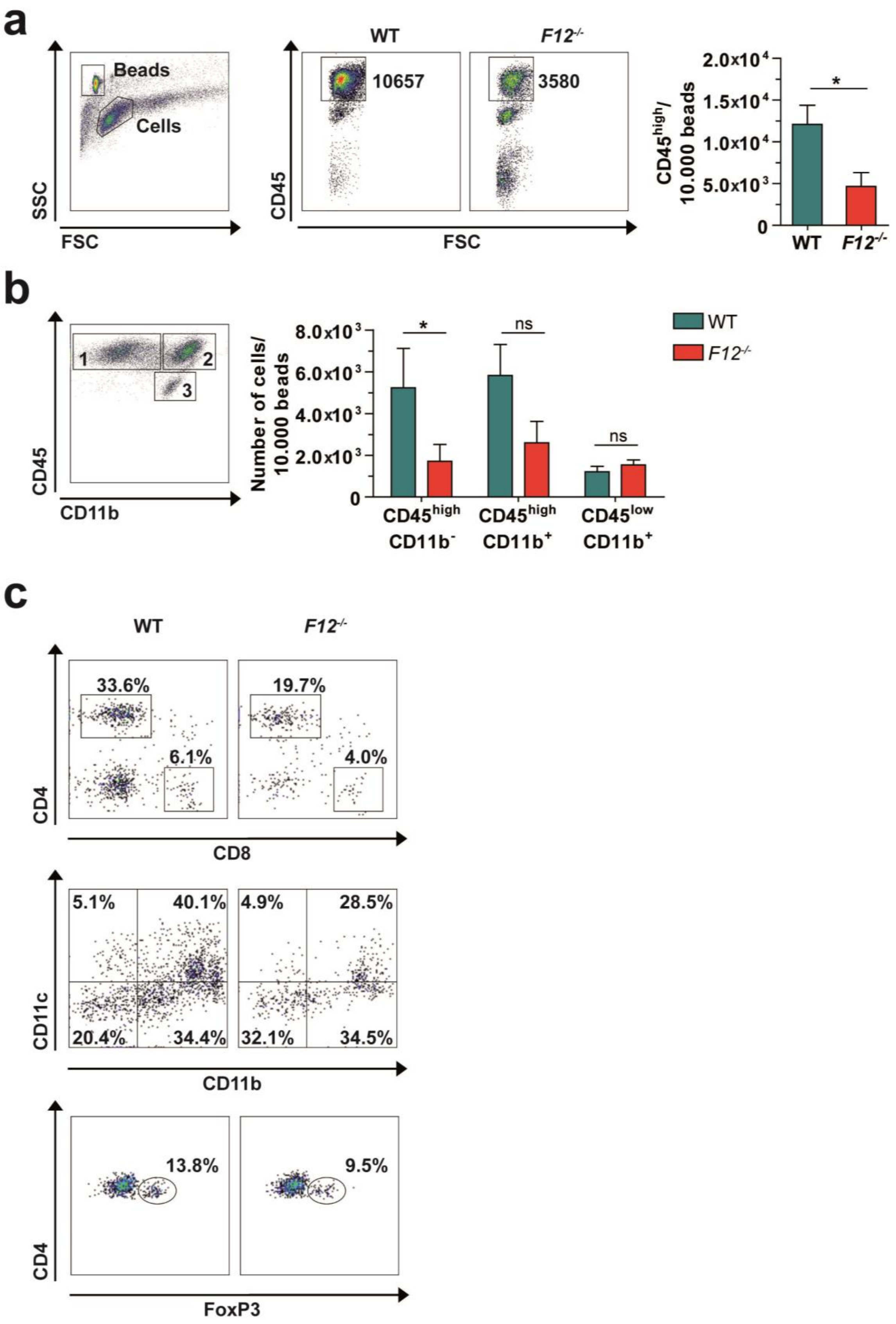

Supplementary Figure 2. Brain-infiltrating leukocytes (BIL) from WT and *F12*$^{-/-}$ mice were analyzed at d_{max} of EAE. BIL isolated from WT and *F12*$^{-/-}$ mice were stained for indicated surface and intranuclear markers before flow cytometric examination. (**a**) Cell surface staining for CD45 allowed assessing the amount of peripheral infiltration into the CNS at d_{max} (day 16) of EAE. Thus, the number of CD45^{high} cells calculated relative to 1.0 × 10^4 reference beads is shown. (**b**) BIL were further analyzed for the numbers of CD45^{high}CD11b^{-} (1, identifying peripheral lymphocytes), CD45^{high}CD11b^{+} (2, identifying peripheral macrophages and dendritic cells), and CD45^{low}CD11b^{+} (3, identifying microglia) cells relative to 1.0 × 10^4 reference beads. (**c**) Representative dot plots showing the percentages of central nervous system-infiltrating CD4^{+}, CD8^{+}, CD11c^{+}, CD11b^{+}, and CD4^{+}Foxp3^{+} cells. In **a** and **b**, data are given as means ± s.e.m. of two independent experiments with four mice per genotype (non-parametric Mann-Whitney *U*-test). Representative dot plots for indicated markers are displayed. In **c**, data are representative of three independent experiments with three or four mice per genotype. $^{*}P < 0.05$; FSC, forward scatter; ns, not significant; SSC, side scatter.

Supplementary Figure 3

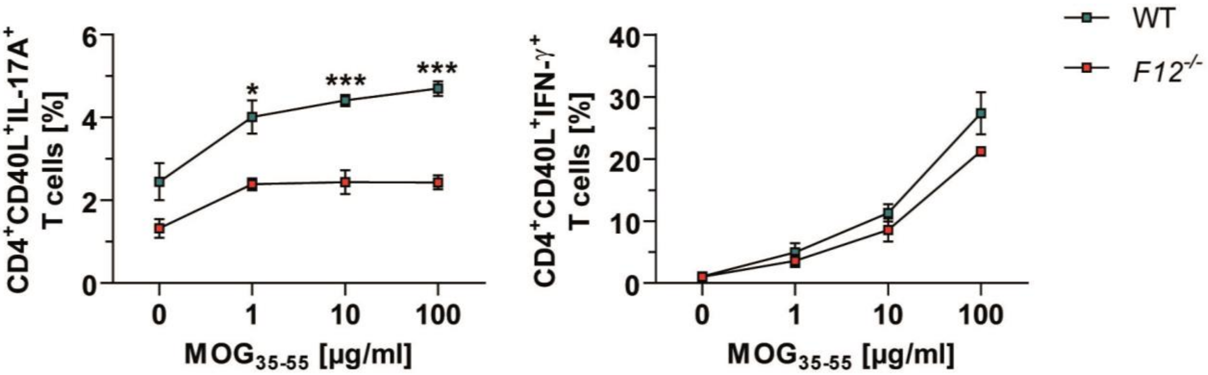

Supplementary Figure 3. Reduced percentages of IL-17A-producing $CD4^+CD40L^+$ T cells at day 10 of EAE. $CD4^+$ T cells (3×10^6) isolated from the LN of WT and $F12^{-/-}$ mice at day 10 of EAE were restimulated with different concentrations of $MOG_{35–55}$. After 2 days, the percentages of IL-17A- and IFN-γ-producing $CD4^+CD40L^+$ T cells were determined by flow cytometry. Data are representative of three independent experiments with three or four mice per genotype (non-parametric Mann-Whitney *U*-test). $^{*}P < 0.05$, $^{***}P < 0.001$.

Supplementary Figure 4

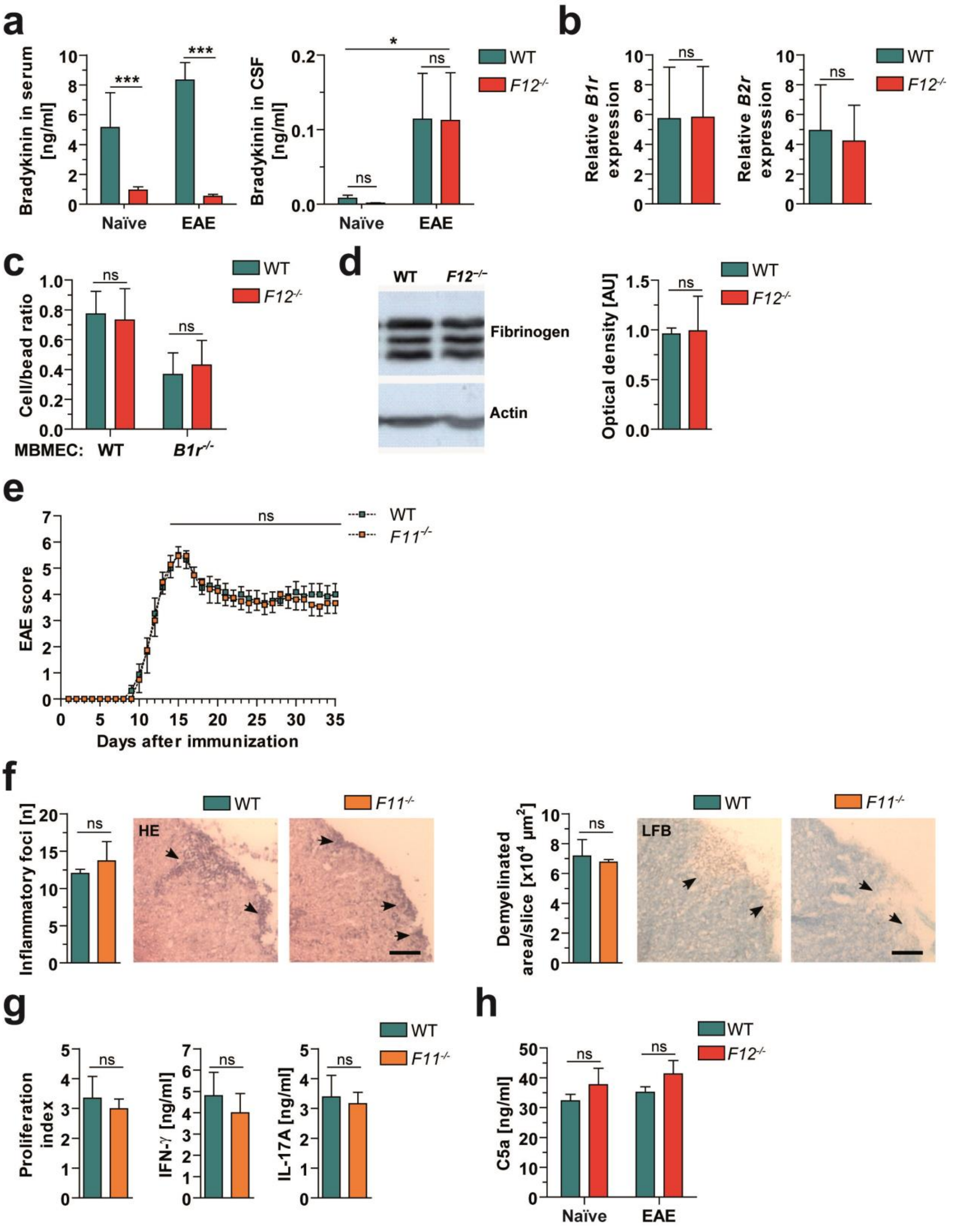

Supplementary Figure 4. Protection from EAE in *F12*$^{-/-}$ mice is independent of the kallikrein–kinin, the complement, or the coagulation system. (**a**) Bradykinin release in serum and CSF of naïve and MOG_{35-55}-immunized WT and *F12*$^{-/-}$ animals at d_{max} (day 16) of EAE. (**b**) *B1r* and *B2r* gene expression in the spinal cords of WT and *F12*$^{-/-}$ mice at d_{max} as determined by rRT-PCR using 18sRNA for normalization. Data are given as fold change in normalized expression in MOG_{35-55}-immunized animals relative to vehicle-injected controls. (**c**) *In vitro* migration of LN cells from WT and *F12*$^{-/-}$ mice across inflamed MBMEC of WT and *B1r*$^{-/-}$ mice. (**d**) Fibrin/fibrinogen deposition, as quantified by optical density measurements, in the spinal cords of WT and *F12*$^{-/-}$ mice at d_{max} of EAE. (**e**) Active EAE was induced in WT and *F11*$^{-/-}$ mice by immunization with MOG_{35-55}. Clinical scores (mean ± s.e.m.) over time of two independent EAE experiments are shown. (**f**) Histological analysis of spinal cord sections from the lumbar region of WT and *F11*$^{-/-}$ animals at d_{max}. Sections were stained with hematoxylin & eosin (HE) to evaluate inflammatory foci (left panel) and immunostained for Luxol fast blue (LFB) to assess demyelination (right panel). Arrows indicate inflammation or demyelinated areas, respectively. Scale bars, 100µm. (**g**) Proliferation and cytokine production by $CD4^+$ T cells purified from LN 10 days after immunization and restimulated with 10 µg ml^{-1} MOG_{35-55} and irradiated (35 Gy) antigen-presenting cells *in vitro* for 48 hours. (**h**) Activation of the complement component C5 to C5a in sera of naïve and MOG_{35-55}-immunized WT and *F12*$^{-/-}$ mice at d_{max}. For **a-d**, data are mean ± s.e.m. of three independent experiments, each performed in duplicate or triplicate. For **f-h**, data are presented as mean ± s.e.m. of two independent experiments. For **a-h**, non-parametric Mann-Whitney *U*-test or student *t*-test. $^{*}P < 0.05$, $^{***}P < 0.001$; AU, arbitrary units; ns, not significant.

Supplementary Figure 5

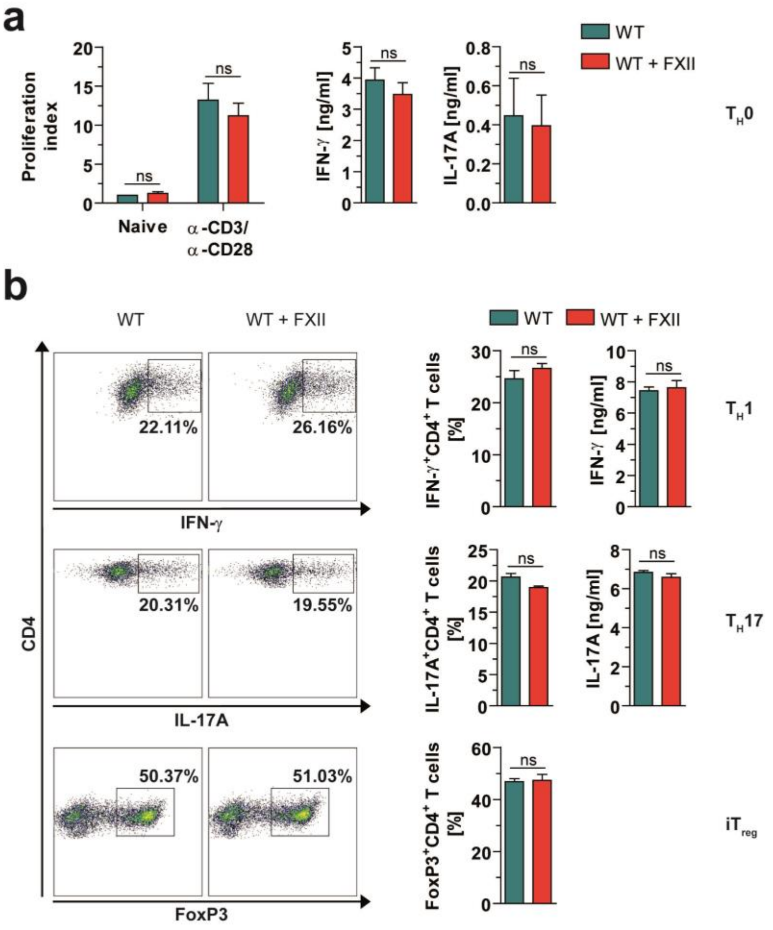

Supplementary Figure 5. CD4$^+$ T-cell polarization is unaltered in the presence of FXII. (**a**) Naïve CD4$^+$CD62L$^+$ T cells from WT LN were polyclonal activated with 5 µg ml^{-1} plate-bound anti-CD3 and 1 µg ml^{-1} soluble anti-CD28 (T_H0) in the presence or absence of FXII. After 48 hours of polyclonal stimulation, proliferation and production of the indicated cytokines were determined. (**b**) Naïve CD4$^+$CD62L$^+$ T cells were activated with anti-CD3 and anti-CD28 in the presence of different recombinant cytokine/neutralizing antibody mixtures specific for T_H1, T_H17, or inducible T_{reg} (iT_{reg}) (see Methods). In **a** and **b**, data are given as means ± s.e.m. of three independent experiments, each performed in triplicate (non-parametric Mann-Whitney *U*-test; ns, not significant).

Supplementary Figure 6

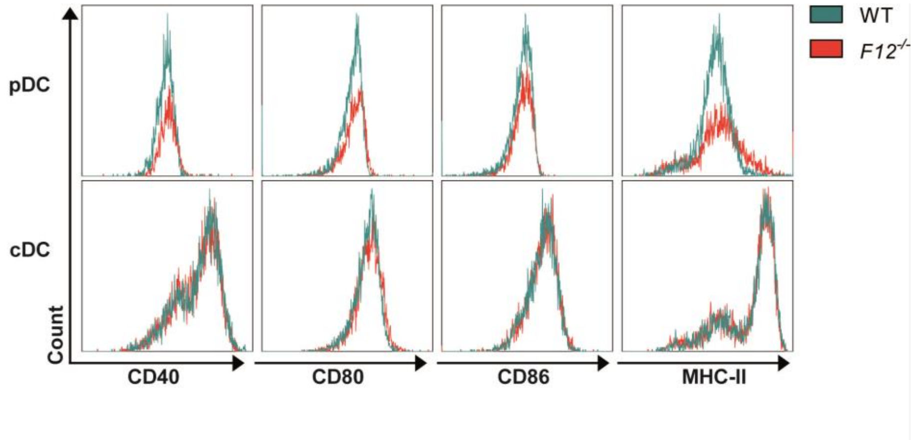

Supplementary Figure 6. DC of *F12*$^{-/-}$ animals have unaltered surface markers. Splenic pDC (upper panel) and cDC (lower panel) of WT and *F12*$^{-/-}$ animals were analyzed for surface expression of the indicated markers by flow cytometry. Data are representative of three independent experiments with three mice per genotype.

Supplementary Figure 7

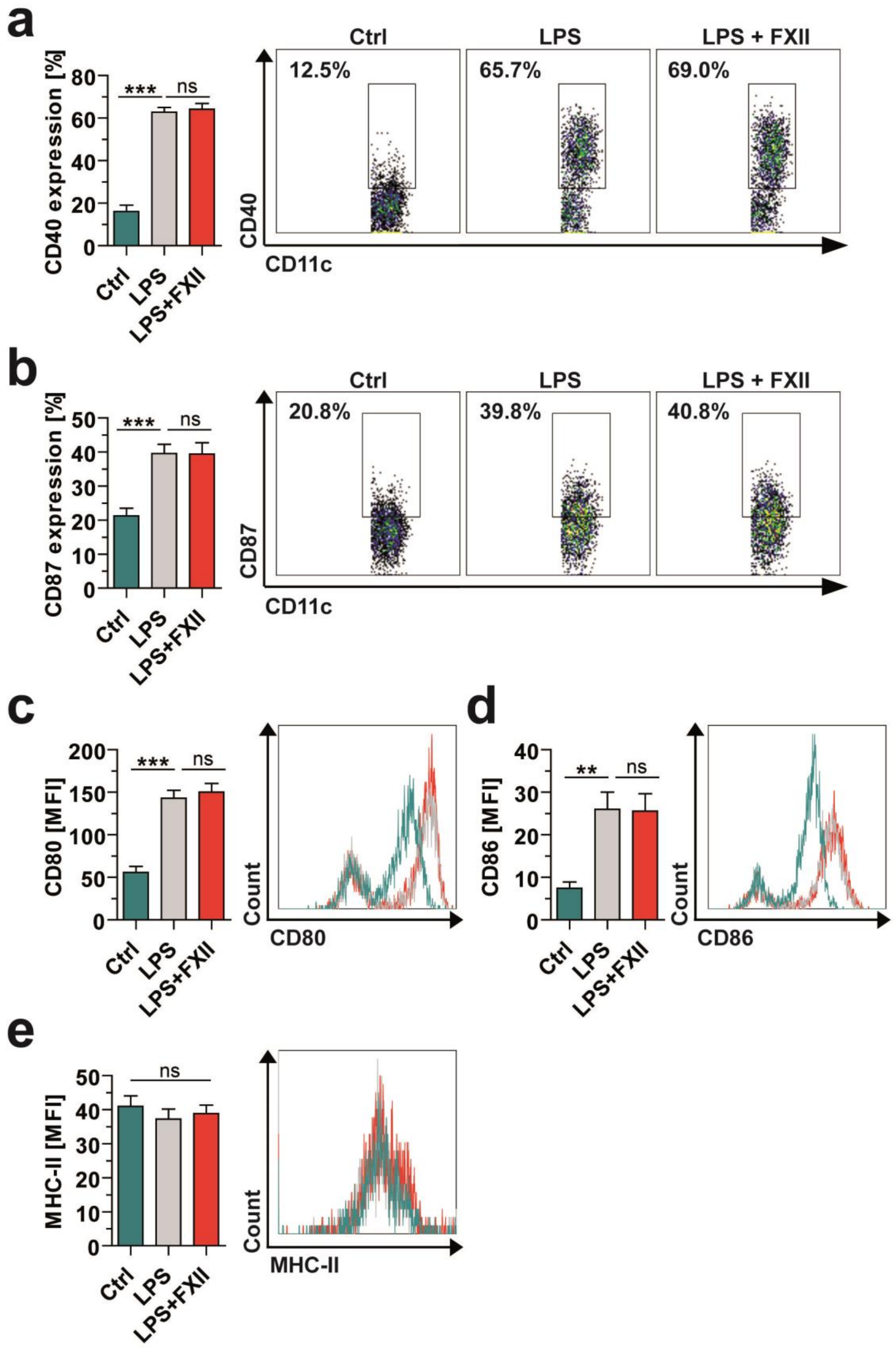

Supplementary Figure 7. FXII has no influence on surface markers of mouse cDC. Splenic cDC of WT animals were incubated with medium only (Ctrl) or stimulated with LPS (1 µg ml^{-1}) in the absence or presence of FXII and analyzed for surface expression of CD40 (**a**), CD87 (**b**), CD80 (**c**), CD86 (**d**), and MHC-II (**e**) by flow cytometry. In **a-e**, quantifications of indicated markers are given as mean ± s.e.m. of three independent experiments with three mice per genotype. Representative FACS plots for indicated markers are also shown. $^{*}P<0.05$, $^{***}P<0.001$ by one way ANOVA and Tukey's multiple comparisons test. MFI, mean fluorescence intensity; ns, not significant.

Supplementary Figure 8

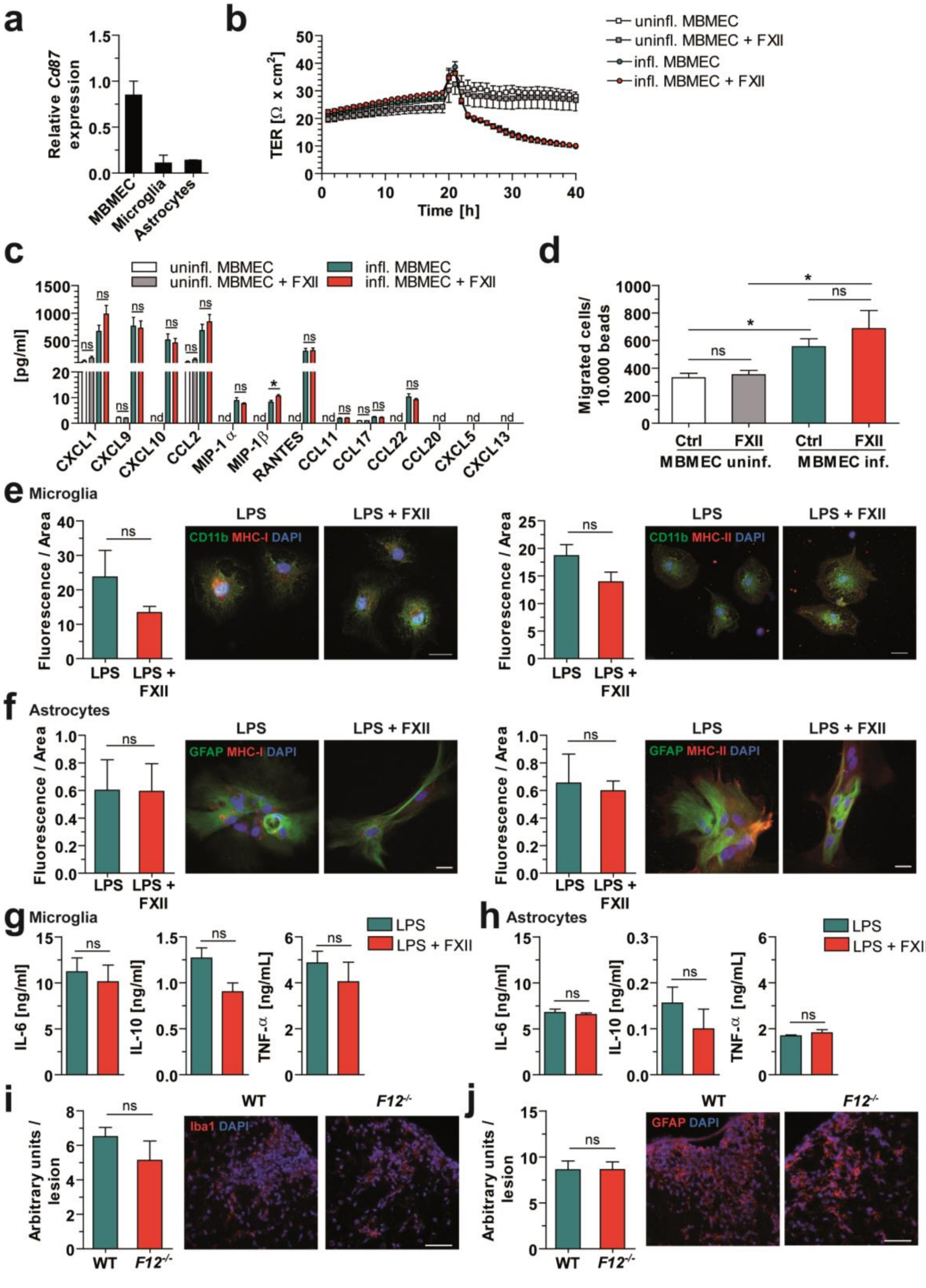

Supplementary Figure 8. FXII has no influence on MBMEC, astrocytes or microglia. (**a**) rRT-PCR analyses for *Cd87* gene expression in primary cell cultures of MBMEC, microglial and astroglial cells are shown. (**b**) The transendothelial resistance (TER) of naïve MBMEC or MBMEC inflamed (infl.) with IFN-γ and TNF-α (each 500 IU ml^{-1}) in the absence or presence of 60 nM factor XII (FXII) was monitored after 24 hours. (**c**) Twenty-four hours post inflammation, MBMEC supernatants were collected and the concentrations of indicated proinflammatory chemokines were assessed by flow cytometry. (**d**) To assess MBMEC permeability, 24 hours post inflammation with or without FXII, naïve splenocytes from wild-type mice were allowed to migrate. The number of migrated cells relative to 1.0×10^4 reference beads was determined for each group by flow cytometry. (**e, f**) Histologic analysis of microglia and astrocyte cultures prior treated with 1 µg ml^{-1} LPS, or 1 µg ml^{-1} LPS and 60 nM FXII for 24 hours. (**e**) Microglia and (**f**) astrocyte cultures were stained with CD11b (green) or glial fibrillary acidic protein (GFAP; green), respectively. Both cell cultures were analyzed for the expressions of MHC-I (left) and MHC-II (right). Scale bars, 20µm. (**g, h**) Production of the indicated cytokines was assessed in supernatants of (**g**) microglia and (**h**) astrocyte cultures after treatment with 1 µg ml^{-1} LPS, or 1 µg ml^{-1} LPS and 60 nM FXII for 24 hours. (**i, j**) Histologic analysis of spinal cord sections from the lumbar region of $MOG_{35–55}$-immunized WT animals at d_{max} (day 16). Sections were stained for (**i**) ionized calcium-binding adapter molecule 1 (Iba1, red) and nucleus (DAPI, blue), or (**j**) GFAP (red) and nucleus (DAPI, blue). Scale bars, 50µm. Data are presented as mean ± s.e.m. of two independent experiments. For **a-j**, Kruskal-Wallis test with Dunn *post hoc* analysis in the case of multiple comparisons or non-parametric Mann-Whitney *U*-test. $^{*}P < 0.05$, CXCL, Chemokine (C-X-C motif) ligand; CCL, chemokine (C-C motif) ligand; MIP, macrophage inflammatory protein; nd, not detected; ns, not significant; uninfl., uninflamed.

Supplementary Figure 9

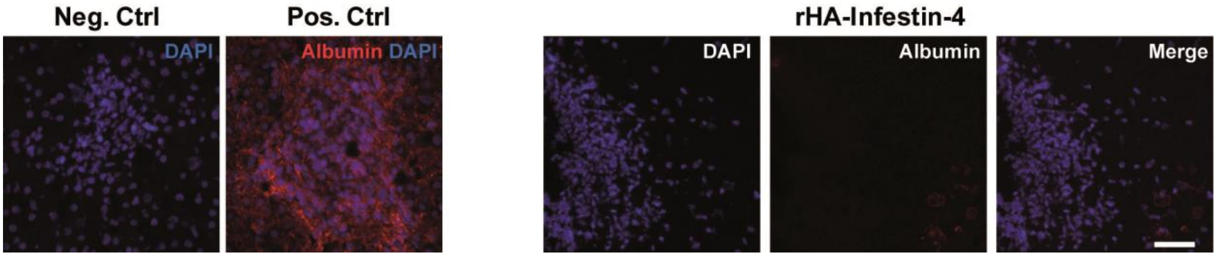

Supplementary Figure 9. **rHA-Infestin-4 linked to human albumin cannot be found in the CNS of EAE mice.** Histologic analysis of human liver (left panel) and spinal cord sections from the lumbar region (right panel) of rHA-Infestin-4-treated EAE mice at d_{max} (day 16). Sections were stained for human albumin (red) and nucleus (DAPI, blue). Scale bar, 50µm.

Supplementary Figure 10

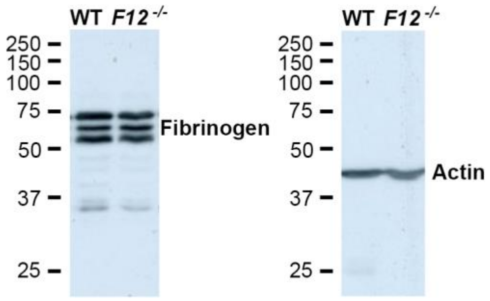

Supplementary Figure 10. Full scan of western blot experiment are shown. For detailed information see Supplementary Figure 4d.

Supplementary Table 1. Detailed information on EAE experiments with *F12*$^{-/-}$ mice induced with MOG$_{35–55}$

Exp. no.	Mice	*n*	Incidence (%)	Drop out (%)	Day of onset (mean ± s.e.m.)	Maximum score (mean ± s.e.m.)
1	WT	9	100	11.1	12.1 ± 0.6	5.4 ± 0.3
1	*F12*$^{-/-}$	7	100	0	14.1 ± 0.7	3.1 ± 0.6
				P = 0.347	*P* = 0.030	*P* = 0.011
2	WT	6	100	16.7	11.7 ± 0.3	5.7 ± 0.2
2	*F12*$^{-/-}$	10	90	0	13.8 ± 0.6	3.5 ± 0.7
				P = 0.363	*P* = 0.033	*P* = 0.018
3	WT	8	100	12.5	11.4 ± 0.4	6.0 ± 0.4
3	*F12*$^{-/-}$	10	90	0	15.2 ± 0.8	3.6 ± 0.5
				P = 0.350	*P* = 0.001	*P* = 0.004

Exp., Experiment; s.e.m., standard error of the mean.

Supplementary Table 2. Immune cell distribution in spleens, LN and brain-infiltrating leukocytes (BIL) of naïve (−) and $MOG_{35–55}$-immunized (+) in WT and *F12*$^{−/−}$ animals

	Spleen				LN				BIL			
Mouse strain	WT		*F12*$^{−/−}$		WT		*F12*$^{−/−}$		WT		*F12*$^{−/−}$	
$MOG_{35–55}$	−	+	−	+	−	+	−	+	−	+	−	+
Cell phenotype												
% of total viable cells												
$CD4^+$	17.7	12.6	17.4	11.1†	21.1	15.9	21.0	15.3	nd	6.6	nd	0.7§
$CD8^+$	12.6	9.5†	12.7	8.2	16.4	14.4	18.7	14.0	nd	0.9	nd	0.3§
$\gamma\delta^+$	2.2	1.4*	1.8	1.5	0.8	0.6	0.6	0.9	nd	0.3	nd	0.4
$B220^+$	45.9	38.3†	48.6	38.6†	18.3	33.4†	18.2	31.2†	nd	5.5	nd	6.6
$CD11b^+$	5.6	28.7*	5.6	27.8*	nd	nd	nd	nd	nd	14.0	nd	1.7§
$CD11c^+$	3.9	2.6	2.6§	2.4	5.7	5.6	5.2	5.9	nd	5.0	nd	0.9§
% of gated $CD4^+$ cells												
$CD25^+$	8.6	7.3	9.6	8.0	11.0	12.8	12.2	13.7	nd	21.5	nd	15.2§
$Foxp3^+$	7.1	6.9	7.8	7.9	9.6	11.8†	11.8§	12.8	nd	12.4	nd	9.2

Nd, not detected. *, $P < 0.01$ (EAE vs. respective control), †, $P < 0.05$ (EAE vs. respective control), §, $P < 0.05$ (*F12*$^{−/−}$ vs. WT)

Supplementary Table 3. Detailed information on EAE experiments under rHA-Infestin-4 treatment in C57BL/6 mice immunized with $MOG_{35–55}$

Exp. no.	Treatment	*n*	Incidence (%)	Drop out (%)	Day of onset (mean ± s.e.m.)	Maximum score (mean ± s.e.m.)
1	Vehicle	8	100	12.5	8.6 ± 0.4	6.5 ± 0.1
1	rHA-Infestin-4	8	100	0	10.3 ± 0.5	5.1 ± 0.2
				P = 0.351	*P = 0.031*	*P = 0.033*
2	Vehicle	10	100	10.0	11.4 ± 0.4	6.3 ± 0.3
2	rHA-Infestin-4	8	90	0	12.8 ± 0.4	4.0 ± 0.6
				P = 0.343	*P = 0.036*	*P = 0.012*

Exp., Experiment; s.e.m., standard error of the mean.

Supplementary Table 4. Detailed information about EAE under rHA-Infestin-4 treatment in SJL/JRj mice immunized with proteolipid protein peptide 139–151

**Mice**	**Treatment**	***n***	**Incidence (%)**	**Drop out (%)**	**Day of onset (mean ± s.e.m.)**	**First attack score (mean ± s.e.m.)**	**Second relapse score (mean ± s.e.m.)**
SJL	Vehicle	9	100	0	8.8 ± 0.2	5.1 ± 0.7	4.0 ± 0.2
SJL	rHA-Infestin-4	9	100	0	8.6 ± 0.2	5.1 ± 0.6	2.1 ± 0.4
					P = 0.619	*P = 0.879*	*P = 0.002*

S.e.m., standard error of the mean.

Supplementary Table 5. Demographic information on patients and HD included in the study

	**CIS**	**RRMS**	**PPMS**	**SPMS**	**HD**
Age (range), years	33.5 (25−51)	37.1 (22−60)	54.0 (44−73)	52.7 (34−72)	45.3 (25−63)
Male/female	6/13	35/103	6/7	32/58	39/91
EDSS	1.5 (0−3.0)	2.5 (0−6.5)	4.5 (2.5−7.5)	5.5 (2.5–8.0)	−
No therapy	16	49	5	17	−
Azathioprine	−	−	−	23	−
Fingolimod	−	25	−	−	−
Glatiramer acetate	1	9	−	−	−
Interferon	2	27	−	8	−
Mitoxantrone	−	3	3	12	−
Natalizumab	−	25	−	−	−
Steroids	−	−	5	30	−

Unless otherwise stated, values are the number of individuals. EDSS, Expanded Disability Status Scale.
